# BiomeNet: A Bayesian Model for Inference of Metabolic Divergence among Microbial Communities

**DOI:** 10.1371/journal.pcbi.1003918

**Published:** 2014-11-20

**Authors:** Mahdi Shafiei, Katherine A. Dunn, Hugh Chipman, Hong Gu, Joseph P. Bielawski

**Affiliations:** 1 Department of Mathematics & Statistics, Dalhousie University, Halifax, Nova Scotia, Canada; 2 Department of Biology, Dalhousie University, Halifax, Nova Scotia, Canada; 3 Department of Mathematics & Statistics, Acadia University, Wolfville, Nova Scotia, Canada; Heinrich Heine University, Germany

## Abstract

Metagenomics yields enormous numbers of microbial sequences that can be assigned a metabolic function. Using such data to infer community-level metabolic divergence is hindered by the lack of a suitable statistical framework. Here, we describe a novel hierarchical Bayesian model, called BiomeNet (Bayesian inference of metabolic networks), for inferring differential prevalence of metabolic subnetworks among microbial communities. To infer the structure of community-level metabolic interactions, BiomeNet applies a mixed-membership modelling framework to enzyme abundance information. The basic idea is that the mixture components of the model (metabolic reactions, subnetworks, and networks) are shared across all groups (microbiome samples), but the mixture proportions vary from group to group. Through this framework, the model can capture nested structures within the data. BiomeNet is unique in modeling each metagenome sample as a mixture of complex metabolic systems (metabosystems). The metabosystems are composed of mixtures of tightly connected metabolic subnetworks. BiomeNet differs from other unsupervised methods by allowing researchers to discriminate groups of samples through the metabolic patterns it discovers in the data, and by providing a framework for interpreting them. We describe a collapsed Gibbs sampler for inference of the mixture weights under BiomeNet, and we use simulation to validate the inference algorithm. Application of BiomeNet to human gut metagenomes revealed a metabosystem with greater prevalence among inflammatory bowel disease (IBD) patients. Based on the discriminatory subnetworks for this metabosystem, we inferred that the community is likely to be closely associated with the human gut epithelium, resistant to dietary interventions, and interfere with human uptake of an antioxidant connected to IBD. Because this metabosystem has a greater capacity to exploit host-associated glycans, we speculate that IBD-associated communities might arise from opportunist growth of bacteria that can circumvent the host's nutrient-based mechanism for bacterial partner selection.

## Author Summary

Metagenomic studies of microbial communities yield enormous numbers of gene sequences that have a known enzymatic function, and thus have potential to contribute to community-level metabolic activities. Ecologically divergent microbial communities are presumed to differ in metabolic repertoire and function, but detecting such differences is challenging because the required analytical methodology is complex. Here, we present a novel Bayesian model suitable for this task. Our model, BiomeNet, does not assume that microbiome samples of a certain type are the same; rather, a sample is modeled as a unique mixture of complex metabolic systems referred to as “metabosystems”. The metabosystems are composed of mixtures of subnetworks, where subnetworks are mixtures of reactions related by function. Application of BiomeNet to human gut metagenomes revealed a metabosystem with greater prevalence among IBD patients. We inferred that this metabosystem is likely to be closely associated with the human gut epithelium, resistant to dietary interventions, and interfere with human uptake of an important antioxidant, possibly contributing to gut inflammation associated with IBD.

This is a *PLOS Computational Biology* Methods article.

## Introduction

Microorganisms comprise up to one-third of the Earths' biomass, and those living on a human can outnumber their cells by a factor of ten [1]. These microbes are believed to form metabolically integrated communities [2] playing a critical role at many levels, from globally significant nutrient cycling [3] to influencing human physiology [4], [5]. Because much of this diversity cannot be cultured in the laboratory, these systems remain largely unstudied [6], [7]. Recent advances in sequencing technology allow access to these communities through sequencing of DNA as it exists in the natural environment (metagenomics) [8]. Several ambitious projects are dedicated to filling the large gap in knowledge through massive sampling, sequencing and analysis of microbiome data. Among their goals is to determine the extent to which different microbiomes share core functions, and to identify associations between changes in microbiomes and changes in complex systems ranging from climate (*e.g*., the Earth Microbiome Project [9]) to human physiology (*e.g*., the Human Microbiome Project [10]). Considerable effort has been directed to developing analytical tools for these data [11]. Notably absent, however, is a model-based framework for analysing metabolic interactions according to the enzyme abundance information within microbiomes.

High-throughput shotgun sequencing yields enormous numbers of environmental DNA sequences, some being homologous to genes known to encode an enzyme and thus assignable to a metabolic function. Development of dedicated analytical methods for these data lags behind the capacity to generate it. The more popular strategies, although yielding promising results, highlight the analytical challenges. First, functional differences among microbiomes often stem from differing abundances of shared metabolic functions rather than presence-absence polymorphisms [12]. Second, one-by-one analysis of enzyme abundance, a very common approach, neglects the non-independence relations among reactions functioning as a network [13] and thus hinders discovery of metabolic functional units and their interactions. Third, each independent analysis of enzyme abundance has restricted power by limiting the analysis to a small part of the data. Although several methods have been developed for analysing the community-wide patterns of metabolic interactions [14]–[17], none employ a formal stochastic model for networks. Jiao et al. [18] developed a novel probabilistic approach to profiling community metabolic function that focuses on metabolic reactions rather than individual genes. Jiao et al. [18] demonstrate that focusing on reactions provides a better representation of the collective metabolic behaviour of microbial communities, and avoids the problem of relying on arbitrary boundaries of annotated pathways. Their method, however, does not utilize enzymes abundances, nor does it formally model multiple microbiome samples as alternative realizations of the constraints that arise from an underlying network structure.

Here, we introduce a Bayesian modelling approach for inferring differential usage of metabolic networks among microbial communities. We approach the problem by considering metabolic structure and function as arising from overlapping metabolic phenotypes referred to as “metabosystems”. A metabosystem is further characterized by a mixture of overlapping metabolic “subnetworks” (*e.g*., sets of reactions related by function). In order to discover metabosystems and subnetworks, and group microbial communities according to differential usage of the metabosystems and subnetworks, we propose BiomeNet, a novel mixed-membership statistical model for metabolic network data. The model takes the reaction abundance data for each metagenome sample as its input. Reactions and their abundances are derived from the abundance of enzyme-encoding gene sequences found within shotgun metagenomic datasets. Suitable datasets are now readily available from public resources such as MGRAST [19] and MetaHIT [20]. Our approach to the analysis of enzyme abundance information is unique in that (i) it works directly with the network data, (ii) reactions within a microbiome are not independent, (iii) contributions of reactions to a subnetwork do not rely on human annotation (*e.g*., a subnetwork might be comprised of parts of different KEGG pathways), (iv) differential usage of subnetworks is modelled through metabosystem composition, and (v) samples with the same typological labels (*e.g*., individuals having a certain disease) need not be identical; rather, the full community metabolism of a sample can exist as a mixture of different metabosystems.

BiomeNet is fundamentally different from other unsupervised methods including Principal Component Analysis (PCA) and its variants. BiomeNet takes advantage of dependencies between reactions encoded by metabolic networks without any data transformation and reduction. Moreover, BiomeNet is specifically designed to provide explanatory capabilities. Differentiating metabosystems discovered by BiomeNet can be explained by their differential usage of metabolic subnetworks, which in turn are interpretable as a small set of strongly connected metabolic reactions. Because interpreting principal components discovered by PCA is not trivial [21], BiomeNet provides a valuable addition to methods such as PCA that are widely used to analyse metagenomic variation. Metabolic networks can also be used to study the relative change in the production or consumption of specific metabolites [22]. Our model, however, is intended to provide a framework for explaining metabolic divergence across microbiome samples as a function of tightly connected subnetworks having different abundances. Like Jiao et al. [18] we prefer to work with reactions rather than individual enzymes. By modelling reaction abundances, we permit promiscuous enzymes (which can catalyse several different reactions) to contribute to the signal for differential usage of subnetworks among different microbiome samples.

We validate our inference algorithm via simulation and apply it to datasets from two studies of functional divergence among gut microbiomes [13], [20]. Gut microbiomes have an intimate physiological interaction with their host, playing an important role in absorption of nutrients, modulation of the immune system and protection against invasion by antagonistic microbes [4], [5]. We focus our analyses on differences between mammals with different dietary niche-types [13], and between healthy and inflammatory bowel disease (IBD)-afflicted humans [20]. In each dataset, we identify “core” subnetworks; these are abundant within each microbiome sample and are not discriminatory. We also resolve discriminatory subnetworks; these are differentially abundant in one or more of the metabosystems. We illustrate how BiomeNet allows us to estimate and represent a sample as a mixture of different types of metabolically-integrated communities. Lastly, we show how the discriminatory subnetworks uncovered by our model provide insight into the biological basis of divergence between different microbiome samples.

## Methods

### Motivation and overview of the analytical framework

BiomeNet is a hierarchical mixed-membership model, where metabolic reactions are mixed to form subnetworks, subnetworks are mixed to form metabosystems, and environmental samples are treated as potential mixtures of metabosystems. We explicitly model metabolism as a hierarchy because biochemical networks are widely considered to be organized in this way. For example, the KEGG pathways database arranges biochemical reactions into sub-pathways (consecutive reaction steps within curated pathways), which are arranged into “pathway modules” that are intended to represent functional units [23]. Alternatively, analyses of metabolic interactions from a purely topological perspective also support the view of hierarchical modularity [24]–[26]. We differ, however, in desiring a probabilistic framework for discriminating the structural components of a full biochemical network. Our mixed-membership approach permits the components of one level (*e.g*., reactions) to contribute to other structures to different degrees (*e.g*., to different subnetworks, metabosystems and samples). Aside from avoiding the need to place arbitrary boundaries on the components of a biochemical network, this framework reflects how community-wide metabolic activities of a microbiome arise from mixtures of organisms having different metabolic repertoires [12], [14], and how microbiome samples are often comprised of mixtures of different communities (*e.g*., stool samples are comprised of mixtures of the epithelium and luminal niche communities [27]).

**Figure 1:**
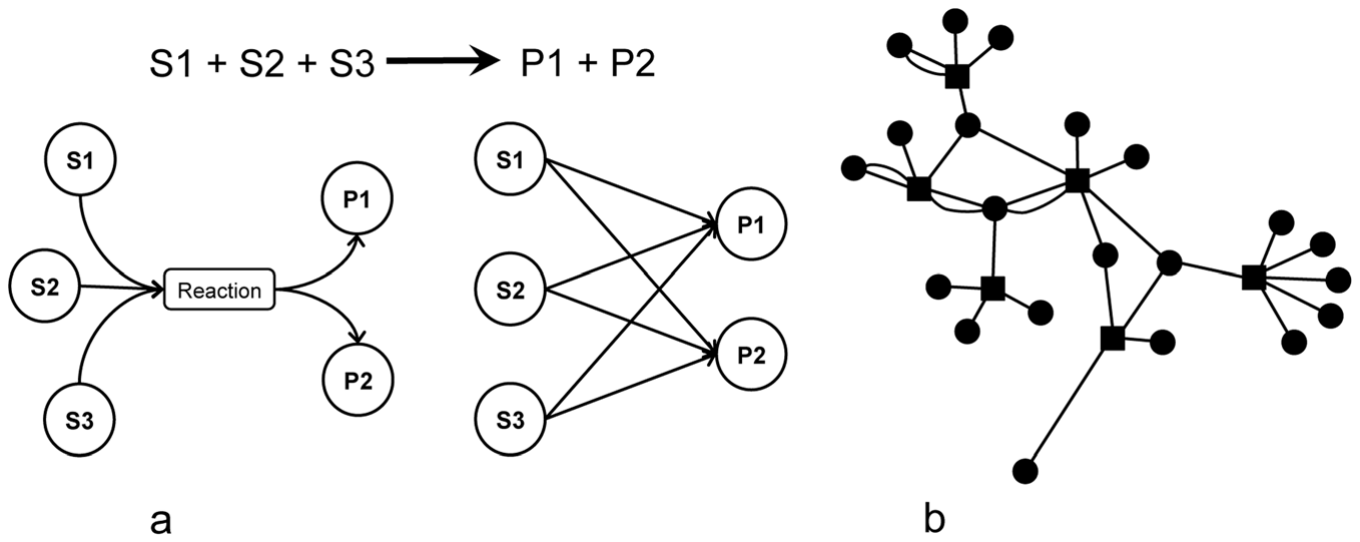
Enzymatic reactions are decomposed into substrate-product pairs that link reactions within metabolic subnetworks. (**a**) An enzymatic reaction is broken down into pairwise relations between its substrates and products. These reactions are more complex than pairwise edges studied in most types of network analysis. Here, reactions are “hyper-edges”, meaning that the relations are between two sets of nodes instead of a pair of nodes. For an undirected reaction, one can consider both directions when breaking the reaction into substrate-product pairs. (**b**) A subnetwork is composed of a set of reactions. In the model, different subnetworks can potentially share reactions and therefore have overlapping regions.

Within BiomeNet, each enzymatic reaction is decomposed into substrate-product pairs (Figure 1a). Dependence among pairs is modeled through shared membership in a subnetwork comprised of substrates and products related by reactions. Because compounds can serve as both substrate and product, reaction pairs within a subnetwork are non-independent (Figure 1b), they are only independent once conditioned on their subnetwork assignment. Subnetworks are not rigidly defined in the model. Each reaction could have some membership to any of the subnetworks. However, for model identifiability and interpretability, we want subnetworks to be defined by a relatively few major reactions, with all other reactions having a negligible membership. Therefore, we model membership in subnetworks as sparse probability vectors. This also permits subnetworks to partially overlap, with some reactions participating in several subnetworks with different degrees.

Metabosystems are modeled as a mixture of *L* subnetworks each contributing to different degrees. We model their composition with a mixture variable *φ*. For example, if we assume that we have 10 subnetworks (*L* = 10), we can look at metabosystem *k* and see it has mixing probabilities for each subnetwork *φ_k_*  =  (≪0.001, ≪0.001, 0.2, ≪0.001, ≪0.001, 0.1, ≪0.001, ≪0.001, 0.7, ≪0.001). We can see from this example that metabosystem *k* is mainly comprised of 20% subnetwork 3, 10% subnetwork 6 and 70% subnetwork 9.

Finally, each microbiome sample is modeled as a mixture of metabosystems. These metabosystems can be thought of as different facets of community-level metabolic activities. Considering the case of gut microbiome samples from diseased and healthy individuals, it is unlikely that the community metabolism of all diseased individuals will be exactly the same. Individuals with a more severe case of the disease might have a larger contribution from a “dysbiotic metabosystem” to their microbiome. Thus, we do not assume that samples are necessarily comprised of just one metabosystem. We denote the metabosystem mixture for a sample as *θ*. For example, if we have 3 metabosystems (*K* = 3), and a microbiome sample has a metabosystem mixture of *θ* = (0.2, ≪0.001, 0.8), then *θ* indicates that this sample consists of 20% metabosystem 1 and 80% metabosystem 3.

Our model is completely unsupervised; the membership of reactions to subnetworks, the contribution of subnetworks to metabosystems, and the mixture of metabosystems within a sample are learned from the data. Collapsed Gibbs sampling [28], [29] is used to infer the posterior distributions of the metabosystem and subnetwork assignments.

### A hierarchical mixed-membership model for BiomeNet

Suppose we have a total of *N* microbiome samples in the data, and those data are comprised of a total of *I_n_* reactions in the *n* ^th^ microbiome sample, and a total of *J_ni_* substrate-product pairs in the *i* ^th^ reaction of the *n* ^th^ sample. Because we expect that a microbiome sample could be a mixture of partially overlapping assemblages of microbes with varying types of ecological interaction, we model each sample as a mixture of *K* metabosystems, where *K* is assumed to be known and fixed in advance. The relative contribution of each metabosystem to the microbiome associated with the *n^th^* sample is modeled through latent variable *θ_n_*, a probability vector of *K* values summing to one. Thus, we have



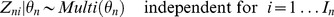
Where, *Z_ni_* denotes the metabosystem assignment for the *i* ^th^ reaction in the *n* ^th^ sample. The *θ* variables will be inferred from the data. We assume an independent and identical (iid) sparse symmetric Dirichlet prior on *θ_n_*.



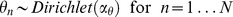
A symmetric Dirichlet is appropriate because we have no prior preference for any of the metabosystems. The probability function of this sparse symmetric Dirichlet distribution is given by
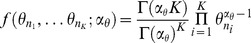
where *α_θ_* is a positive parameter. The mean of 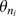
is given by 1/*K*, for *i* = 1,…, *K*, and the variance of 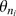 is given by 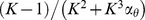. Thus the variance is larger when *α_θ_* is closer to 0, in which case, the probability that a randomly drawn point from this distribution is a sparse vector is larger. The sparsity introduced by a prior can be viewed as equivalent to the penalty added to the likelihood in the penalized likelihood method [30], which here solves the model identifiability issues, reduces the model variance and also improves the model interpretability.

Next, we assume each metabosystem is comprised of a fixed number (*L*) of metabolic subnetworks. Therefore, metabosystems differ with respect to their mixture probabilities of the different subnetworks. The contribution of each subnetwork to the *k^th^* metabosystem, *φ_k_*, is modeled by a vector of *L* mixing probabilities that sum to one. For *K* metabosystems, there will be *K* probability vectors of *L* mixing probabilities. Thus element *φ_kl_* in *K* × *L* matrix *φ* represents the relative contribution of subnetwork *l* in metabosystem *k*. Let *Y_ni_* denote the subnetwork assignment for the *i* ^th^ reaction in the *n* ^th^ sample. Then we model *Y_ni_* as:



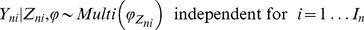
As above, we assume an iid sparse symmetric Dirichlet prior on rows of *φ*.



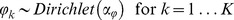
The choice of prior is motivated by the idea that each metabosystem should be mostly a mixture of relatively few subnetworks. For a given metabosystem, subnetworks contributing significantly more to this metabosystem in comparison to the other metabosystems will differentiate this metabosystem from the others and are considered “discriminatory” subnetworks.

Finally, we assume each subnetwork is comprised of a mixture of reactions. This implies that subnetworks differ according to their particular mixture of reactions. One of the goals of the model is to find connected subnetworks of reactions carrying out a function or a set of functions. Therefore, reactions contributing to a subnetwork cannot be considered independent.

Reactions within a subnetwork are linked through their shared chemical compounds. We model subnetworks as a subset of compounds that are converted to another subset of compounds. It is reasonable to assume that the substrate and product sets cannot be arbitrary sets of compounds. Therefore, we assume that compounds are grouped together and make up substrate groups and product groups. Each subnetwork has its own substrate group (*S*) and product group (*R*). The compounds in the substrate group associated with a subnetwork are used as substrates in reactions that belong to that subnetwork. The product group associated with a subnetwork consists of products that are produced by at least one of the reactions in the subnetwork. Note that a compound can belong to both the substrate and the product group. Such a compound will be involved in the intermediary reactions of the subnetwork. In general, membership of a compound to a substrate or product group is considered to be a “soft” (*i.e*., probabilistic) membership.

For *L* subnetworks, we have *L* substrate and *L* product groups modeled as probability vectors over all compounds. For subnetwork *l*, we denote the substrate group as *δ_l_* and the product group as *γ_l_*, each a vector of *C* probability values summing to one, where *C* is the number of compounds. With *L* subnetworks, there are two *L* × *C* matrices *δ* and *γ,* one for substrates and one for products respectively. The value in row *l* and column *c* of matrix *δ* represents the relative contribution of compound *c* in the substrate group of subnetwork *l*. A similar definition applies for product groups in matrix *γ*.

We assume iid sparse symmetric Dirichlet priors for the rows of both matrices. We expect to have a relatively small number of compounds in each subnetwork and therefore, a Dirichlet prior with a small *α* value serves as a proper candidate.



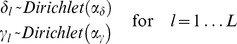
We induce the dependencies between substrates and products by conditioning on their subnetwork membership. Specifically, the substrate-product pairs are considered conditionally independent given the subnetwork membership of the corresponding reaction.

Each reaction *i* in sample *n* is broken down into *J_ni_* substrate-product pairs (Figure 1), with each pair denoted as *S_nij_* → *R_nij_*. Conditioning on the membership of its reaction in the *l* ^th^ subnetwork, the probability of each substrate and product is:



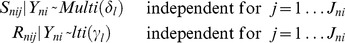
The substrate-product pairs in a reaction are linked through the subnetwork assigned to the reaction.

**Figure 2:**
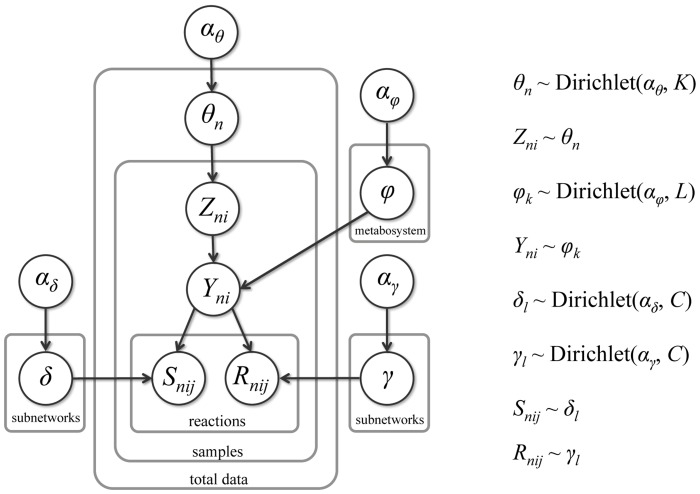
Plate diagram for the BiomeNet model. *θ* is the probability distribution of possible metabosystems in a sample. *Z* represents metabosystems and *Y* represents subnetworks. *φ* is the prior distribution of subnetworks in metabosystems. *δ* is the prior distribution of substrate compounds in subnetworks. *γ* is the prior distribution of product compounds in subnetworks. *α* is the concentration parameter of the Dirichlet distribution. *K* is the number of metabosystems. *L* is the number of subnetworks. *C* is the number of compounds. To indicate relationships, *n* indexes a sample, *i* indexes a reaction, and *j* indexes substrate-product pairs. This model specifies a generative process; coupling between substrate-product pairs is enforced by conditioning their generation on a single subnetwork membership.

BiomeNet is a generative model, and a full description of how to generate a network from it is provided in Text S1. A plate diagram of the model is provided in Figure 2.

### Model inference

The complete likelihood of the data given the hyper-parameters of the prior distributions is:
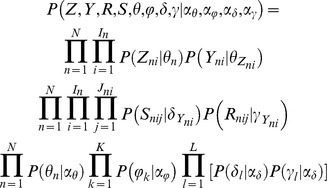
where *Z, Y, θ, φ, δ*, and *γ* are latent variables in our model. *Z* and *Y* collectively represent the metabosystem and subnetwork assignments for all reactions in all samples. Finally, *S* → *R* represent the set of all substrate-product pairs observed in our dataset for all reactions in all samples. To infer the model framework, we need to sample from the posterior distribution of latent variables given the data:
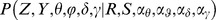
This is a high dimensional distribution having conditional distributions that can be sampled relatively easily. Therefore, we use collapsed Gibbs sampling [29] by integrating out the other latent variables *θ, φ, δ, γ* and sample from the posterior distributions of the metabosystem (*Z*) and subnetwork (*Y*) assignments for each reaction conditional on the assignments of all other reactions.

More specifically, each iteration of Gibbs sampling will provide one sampling point from the joint posterior distribution 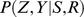. We use the following conditional probability to sample the subnetwork and metabosystem assignment of one reaction in a microbiome sample given we know the subnetwork and metabosystem assignments of all other reactions in every microbiome sample:
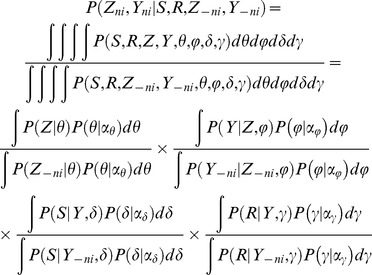
where *Z_-ni_* and *Y_-ni_* denote the metabosystem and subnetwork assignment for all reactions in all samples except only reaction *i* in sample *n*. The individual terms in the above equation can be analytically derived (Text S2). Each iteration of Gibbs sampling cycles through all reactions in all microbiome samples. Thus it will not only provide one sampling point from the joint posterior distribution 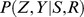, it also provides a sampling point from all marginal distributions 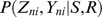.

We can infer the posterior distributions of *θ* and *φ* based on the sampling results for the posterior distribution of *Z* and *Y*. From each iteration of the Gibbs sampling, we get one sampling point of 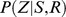, which permits estimation of the *θ* value; *i.e.* for the *n* ^th^ microbiome sample, the estimate of *θ_n_* will be the relative frequencies among all reactions of this microbiome that were assigned to different metabosystems. This estimated *θ_n_* is approximately a sampling point from the posterior distribution 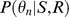. If we take many iteration of the Gibbs sampling results, and estimate *θ_n_* from each iteration, this will provide a sampling distribution for the posterior distribution of *θ_n_*.

For *φ_k_*, the estimate will be relative frequencies of subnetwork assignments among all the reactions that are assigned to the *k* ^th^ metabosystem, where the frequency is taken across all microbiome samples. Similarly, each iteration of Gibbs sampling provides one approximate sampling point from the posterior distribution of *φ_k_*. From the above, the posterior mean of *θ_n_* and *φ_k_* can be directly calculated as the mean of the estimated *θ_n_* and *φ_k_* from many iterations of Gibbs sampling.

BiomeNet's MCMC sampler is implemented in R and C++. Source codes are available from http://sourceforge.net/projects/biomenet/. For both datasets analyzed below, the first 100 samples were considered “burn-in” and were discarded. Following the burn-in, 500 samples were retained, with a lag of 20 iterations of the MCMC between samples.

### Choice of *K*, *L* and hyper-parameters

The purpose of BiomeNet is to learn the structure of the data. It is an unsupervised learning method. As with most other unsupervised learning methods, the validity of the inference is mainly judged through the scientific background of the problem and the interpretability of the results [31]. Here, *K*, is the preset number of “prototypical” structures (metabosystems for reaction data) within the model. Thus, each microbiome sample is interpreted as a mixture of such structures. In unsupervised applications such as those presented in this paper, trying several different *K* values to find the value that shows separation of classes is often considered a natural way to choose *K.* Although this tactic requires some degree of heuristic judgment, typically it is difficult to avoid at least some heuristics in the context of unsupervised learning methods [31].

Ideally, inference under BiomeNet should be largely robust to the choice of *L* as long as *L* is not too small. If *L* is larger than needed, there will be redundant subnetworks in BiomeNet, but they will carry very little weight for any of the metabosystems, and their reactions will have only a trivial impact on the reaction composition of each metabosystem. Alternatively, the informative subnetworks should make consistent contributions to each metabosystem, and their reactions should be highly influential on the reaction composition of each metabosystem. Thus, BiomeNet should be able to infer a consistent reaction profile for each of the *K* metabosystems, as long as the *L* value is not too small. This can be visually verified by using a heat map that portrays differences in reaction composition among metabosystems. The details for computing the reaction composition of a metabosystem, and for comparing the composition of different metabosystems are given in Text S3. In addition to assessing the robustness of reaction composition to the *L* value, the approach can be used to identify a minimum value for *L*.

In our analyses of real data, we chose a value of *K* according to biological criteria. This approach avoids the computational burden associated with assessing many values of both *K* and *L*. However, we can use the same heat map described above to check for divergence among the *K* metabosystems. If there is no signal for divergent metabosystems (*e*.*g*., all the samples have similar, or the same, reaction composition), differences between metabosystems will be similar to those observed for the same metabosystem over different values of *L*. If at least some of the metabosystems are divergent, differences in reaction composition will be large as compared to those within the same metabosystem. In this way, we can assess the chosen value of *K*. Further details are given in Text S3.

The hyper-parameters of our model are the concentration parameters of the symmetric Dirichlet distributions (*i.e.*, *α_θ_, α_φ_, α_δ_*, and *α_γ_*). The values of *α_θ_* and *α_φ_* control the extent by which subnetworks and metabosystems are mixed, while *α_δ_* and *α_γ_* control the size of the subnetworks. If very small values are chosen for *α_δ_* and *α_γ_*, then a relatively larger value of *L* should be chosen. Because we want (*i*) only a few subnetworks to contribute significantly to each metabosystem, and (*ii*) each subnetwork to consist of relatively few reactions, we chose a value for the concentration parameters close to zero (0.01). Users can control the extent of mixing for their data by resetting the concentration parameter values.

## Results

### Simulation and validation of the inference procedure

It is straightforward to simulate data from the model (Text S1). To simulate data, the generative process is repeated for the desired number of microbiome samples. We simulated datasets of different size, with number of samples varying from 40 to 100 by increments of 20. Each sample is associated with a metabolic network generated by the above process. The number of nodes (chemical compounds) for each network varied between 100, 500, and 1000. The number of reactions in each network was assumed to follow a Poisson distribution with mean 1000. The number of substrate-product pairs for each reaction was drawn from a Poisson distribution with mean 2 (plus one to avoid zero). The mixing distribution of metabosystems for samples was drawn from a symmetric Dirichlet distribution with *α* parameter equal to 0.05, 0.10, and 0.20. We simulated datasets with 3 and 5 metabosystems. The subnetwork mixture for each metabosystem was drawn from a symmetric Dirichlet distribution with *α* parameter equal to 0.05, 0.10, and 0.20. This allowed metabosystems to have overlapping subnetworks to different degrees (Text S1). The number of subnetworks varied between 10, 20 and 50.

We fitted BiomeNet using our preferred value for the concentration parameters of the Dirichlet priors of the model (0.01). We compared the estimated mixture weights for metabosystems in each sample with the corresponding weights used for simulation. For each simulation, the inference algorithm was able to recover mixture weights that were very close to the actual metabosystem and subnetwork composition despite fixing value of the concentration parameters at 0.01 (Text S1). Although discrepancies were larger when the misspecification of the concentration parameter was larger, the closeness of the inferred mixture weight indicates robustness of the inference algorithm to the choice of hyper-parameter value within the range examined (0.05 through 0.2).

### Gut communities associated with different mammalian dietary niches are metabolically divergent

Carnivorous, omnivorous and herbivorous mammals are well known for diverse digestive physiologies. Metagenome sequencing of fecal samples from 33 mammal species revealed that 16S communities and metagenomes within the mammalian gut differ according to dietary niche of the host [13]. Based on one-by-one testing of metagenomic sequences, Muegge et al. [13] detected differences in the relative abundance of 495 enzymes between the communities of herbivores and carnivores. However, this approach, in addition to aforementioned issues with one-by-one analyses, provides no information about differences in metabolic subnetworks without post hoc mapping to human-annotated databases. Although applying PCoA reveals separation between carnivore and herbivore gut microbiome (Text S4), PCoA is not equipped with a means to interpret the nature of these differences. We applied BiomeNet to this data, to (*i*) verify that observed differences in gut communities of carnivores and herbivores indeed reflects differences in community metabolic function, and (*ii*) gain insights into the differences in metabolic functions of those communities.

We obtained metagenomic data for 38 samples of mammals [13] as deposited in MGRAST [19] (MGRAST project #116, with the subsystem hierarchical classification). Each sample comprises a separate gut microbiome sample. We extracted gene sequences with an Enzyme Commission (EC) number and recorded their abundance within each sample. The EC number designates the chemical reactions catalyzed by the encoded enzyme, and the reactions were then converted into substrate-product pairs. The fully processed data, formatted for input to BiomeNet, are available with the source code at http://sourceforge.net/projects/biomenet/. This dataset yielded 2,824 unique reactions between 2713 compounds. Reaction abundances for samples, as input into BiomeNet, range from 12,626 to 174,103 (see Text S5 for further detail about how these reaction abundances are obtained from raw reads). We applied BiomeNet to these data assuming that samples could be mixtures of as many as three metabosystems (*K* = 3). The number of metabosystems was initially chosen to match the number of categories for dietary niche (carnivore, herbivore & omnivore). However, because the model does not assign a diet status to a particular metabosystem, they are simply designated as metabosystems 1, 2 and 3, and the uniqueness of their reaction composition was assessed for these data.

**Figure 3:**
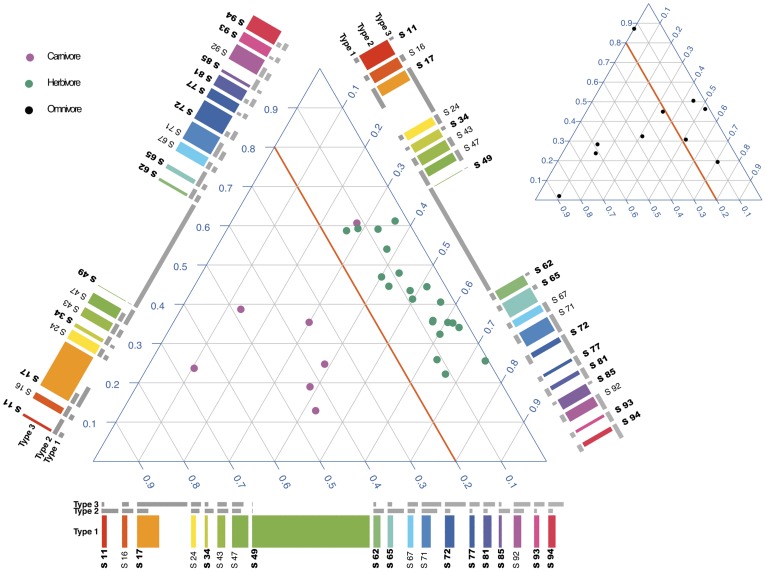
Inferred metabolic composition of carnivore, omnivore and herbivore gut-microbiomes. Mammalian microbiome samples are mapped to three metabosystems and plotted on a simplex (de Finetti diagram). Each point within the simplex represents a sample having a unique set of proportions for the 3 metabosystems. The proportions for each sample sum to one. Lower left, lower right and top corners of the simplex plot indicate 100% membership to metabosystems 1, 2 and 3 respectively. Carnivore (magenta) and herbivore (green) samples are plotted in the large simplex, and omnivore (black) samples are plotted in the small simplex. Each metabosystem is represented in terms of 19 principal subnetworks by using a “composition ribbon” along the sides of the large simplex. Metabosystem 1 and its principal subnetworks are plotted along the horizontal side of the large plot. The colored bars represent the membership of the subnetworks to this metabosystem. The other two sets of grey bars represent the membership of the same subnetworks in the other two metabosystems for comparison. Bold labels indicate the discriminatory subnetworks. One subnetwork, 49, was highly discriminatory for metabosystem 1. It is easy to see from the composition ribbon that subnetwork 49 is substantially more abundant in metabosystem 1 compared to the other two metabosystems. The criteria for selecting principal subnetworks and discriminatory subnetworks are provided in Text S3. Composition ribbon plots for all 100 subnetworks can be found in Text S7.

Using BiomeNet with *L* = 100 subnetworks, we obtained an estimate of the contribution of different metabosystems to each gut community sample. As seen in the simplex plot within Figure 3, carnivore gut communities (magenta dots) tend to have a high membership to metabosystem 1 whereas herbivore gut communities (green dots) tend to have a low membership (*θ_1_*<15%). Interestingly, omnivores did not form a separate cluster; some omnivore communities (black dots) had mixtures more similar to carnivores and others more similar to herbivores (Figure 3). This highlights the benefits of an unsupervised analysis, as compared to an *a priori* (and possibly incorrect) assignment of one metabosystem as unique to omnivores.

**Figure 4:**
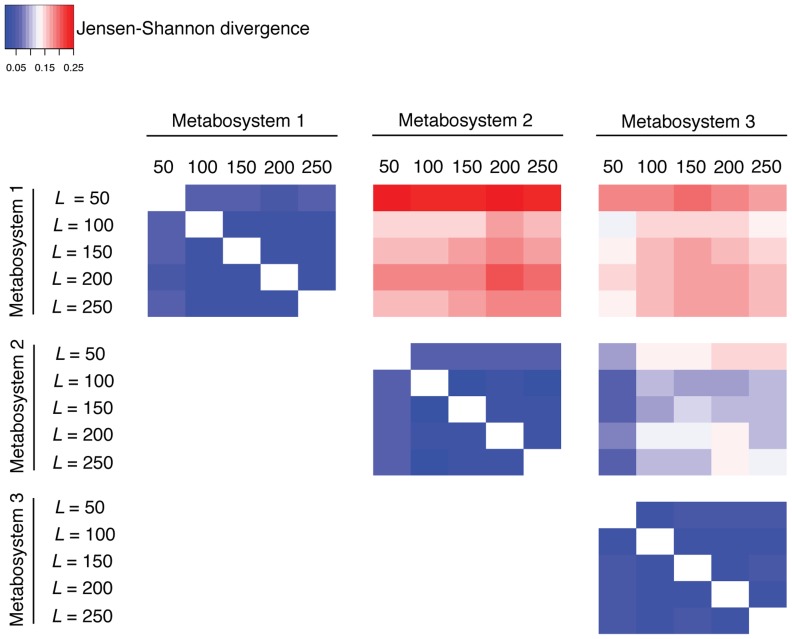
Divergence of reaction composition within and between the three metabosystems inferred from the gut microbiomes of carnivores, omnivores and herbivores. Divergence between metabosystems is measured at the level of their reaction composition by using the Jensen-Shannon divergence (JSD), and presented as a heat map. Further details about computing the reaction composition of a metabosystem are given in Text S3. The JSD score is used here to provide a symmetric measure of the difference between the composition of all 2,824 reactions in these data. The heat map is comprised of six divergence matrices, one for each of the possible pairwise comparisons between the metabosystems. Because the metabosystems had characteristic reaction compositions, it was easy to coordinate metabosystems across different analyses (Text S6). The three matrices along the diagonal represent comparisons within the same metabosystem for different numbers of subnetworks in the model (*L* = 50, 100, 150, 200 and 250); the dominance of blue (low JSD) in those matrices indicates that reaction composition is robust to the *L* value. This result also validates the coordination of those metabosystems into 3 different groups. The three off-diagonal matrices represent comparisons between metabosystems for different numbers of subnetworks (*L*); larger JSD scores here indicate greater divergence between metabosystems.

We investigated the sensitivity of these results to changes in the number of subnetworks (*i.e*., *L* = 50, 100, 150 & 200). Even though the labeling of a metabosystem is arbitrary, they can be distinguished if they have a characteristic composition of reactions. For these data, the metabosystems identified by BiomeNet had different reaction compositions that were largely robust to *L*. This is illustrated by the heat map in Figure 4, which shows the Jensen-Shannon divergence (JSD) [32] between metabosystems according to their reaction composition (see Text S3 for additional details). Within the heat map, divergence matrices along the diagonal represent comparisons within the same metabosystems, and the off-diagonal matrices are for comparisons between metabosystems. Very low JSD (dark blue) along the diagonal matrices in Figure 4 indicates highly similar reaction composition within each of three metabosystem. Higher JSD (pink to red) within both of the off-diagonal divergence matrices that involve metabosystem 1 indicate that there is very strong signal for its uniqueness. JSD scores in the remaining divergence matrix suggest that metabosystems 2 and 3 are also divergent from each other, but not as much as they are from metabosystem 1. When *L* = 50, JSD is consistently lower in the diagonal matrices as compared to *L*>50. Also when *L* = 50, some comparisons between metabosystems 2 and 3 have JSD scores similar to those observed for comparison within the same metabosystem. Taken together, the results indicate that these metabosystems have characteristic reaction compositions, with stable mixture weights in each sample when *K* = 3 and *L*≥100. Consequently, it was easy to coordinate metabosystems across the different analyses (Text S6 and Figures 1 and 2 in Text S6). Thus, all results are hereafter derived from a model with *K* = 3 and *L* = 100, and the mixture probabilities of reactions in all subnetworks are provided in Data File S1 for *K* = 3 and *L* = 100. Note that we are able to separate carnivores and herbivores under a model having *K* = 2, but employ *K* = 3 because there is evidence for samples being a mixture of at least 3 reaction profiles (Figure 4).

Next we used the model to investigate the differences between carnivore and herbivore microbiomes by identifying those subnetworks most diagnostic of metabosystem 1. Recall that metabosystem 1 tended to make a high contribution to carnivore gut communities and low contribution to herbivore gut communities. Rather than attempt to summarize the contribution of all 100 subnetworks to each metabosystem, we show the composition of their “principal subnetworks” via the composition ribbon plots along each side of the simplex (Figure 3). The principal subnetworks are defined as those with a membership>2/*L* to at least one metabosystem (Text S3), and there were 19 principal subnetworks for this dataset. The information is repeated in each ribbon in Figure 3, however using color and increasing the width of the frequency bar emphasizes the contribution of the subnetworks to the selected metabosystem. The grey bars in each ribbon give the relative contribution of subnetworks to the non-selected metabosystems. Among the principal subnetworks, 10 had probability differences large enough to be discriminatory for the metabosystems (Text S3 and S7). Among those 10, subnetwork 49 was highly discriminatory for metabosystem 1 (metabosystem 1 is emphasized in the ribbon along the bottom of the simplex in Figure 3). The ribbon shows how subnetwork 49 makes a large contribution to metabosystem 1 (having a large green bar), but very little contribution to the other two (very small grey bars adjacent to the large green bar). Other discriminatory subnetworks can be seen within the composition ribbon; *e.g*., subnetwork 11 makes a larger contribution to metabotye 2 than to metabosystem 1, and subnetwork 17 makes a larger contribution to metabosystem 3 than to metabosystem 1. Although subnetwork 49 is highly discriminatory, it should not be viewed as a presence-absence polymorphism; each sample is a mixture of metabosystems, with herbivores characterized as having a low, but not zero, contribution of subnetwork 49 to their samples.

**Figure 5:**
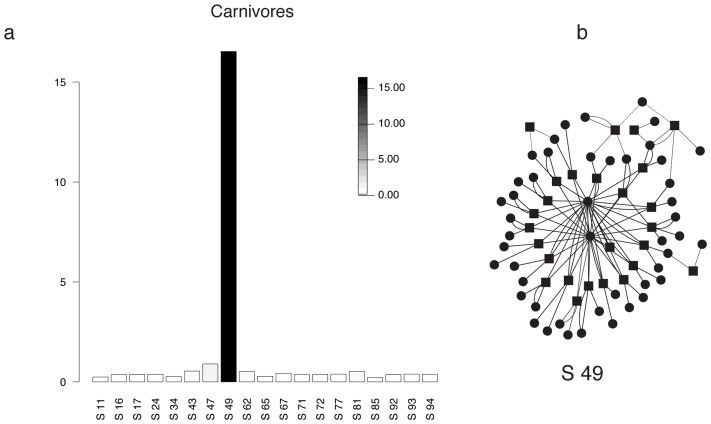
Discriminatory subnetworks for the carnivore associated metabosystem. The histogram in (**a**) gives the relative membership score of subnetworks in metabosystem 1 relative to metabosystems 2 and 3. Membership scores are for metabosystem 1 because it makes a high contribution to carnivore gut communities and a low contribution to herbivore gut communities. The membership score for a subnetwork in a selected metabosystem is based on the ratio of the probability of membership in this metabosystem to its largest probability membership in the other two metabosystems. The score is the absolute value of the logarithm of this ratio. Only the “principal” subnetworks are plotted for carnivores and herbivores. Principal subnetworks for this dataset are defined as subnetworks with a membership>2/*L* to at least one metabosystem. A plot of all 100 subnetworks is provided in Text S7. Note that relative membership scores are computed solely for the purpose of visual assessment of metabosystem composition; the individual value of this score should not be attributed any additional meaning. (**b**) Discriminatory subnetwork 49 is depicted as a network. This network illustrates a high degree of connectivity. Reactions are shown as square nodes and compounds are plotted as circular nodes. Links originating or ending in so-called currency compounds are represented with dashed lines.

Subnetwork 49 stands out because it contributes to metabosystem 1 nearly as much as all other subnetworks combined (Figures 3 & 5 and Text S7). The KEGG reaction numbers, reactant numbers and pathways, as well as mixing probabilities, are given for the principal reactions in Table S1. This subnetwork is dominated by a particular metabolic function: reactions related to importation of extracellular saccharides (N-acetylmuramic acid, N-acetylglucosamine, fucose, glucose and mannose). This suggests that community metabolic function within the carnivore gut might be impacted by their low carbohydrate diet. In particular, the carnivore community appears to be exploiting alternative carbohydrate sources, such as of the cell walls of the gut bacteria themselves, whose outer membranes are composed of alternating molecules of N-acetylmuramic acid and N-acetylglucosamine [33], [34]. Presumably, the dead bacteria in the large intestine comprise a good source of these two compounds. Another nutrient source is the fucosylated mucins secreted by the host's large intestine [35], [36]. Indeed, experimental studies demonstrate that fucose can serve as an important carbon source for at least some species within the mammalian gut, especially under nutrient deprivation [*e.g.*, 37]. Taken together, our results support the recent finding of Koropatkin and co-authors [27] that a high protein diet, such as that found in carnivores, could select for species exploiting the alternative nutrient source represented by mucus glycans. Whatever the source, the carnivore community appears to be exploiting input nutrients that are less important to the gut community of herbivores.

### IBD is associated with a metabosystem having greater capacity to exploit host-associated glycans and interfere with the host capacity to manage oxidative stress

We illustrate the value of our approach to human microbiomics by applying it to a sample of adults who are classified either as healthy or as having IBD. The dataset consists of *N* = 124 adult human gut microbiome samples compiled and sequenced by Qin and co-authors [20]. They found that IBD patients (Crohn's and ulcerative colitis) could be differentiated from healthy individuals according to differences in the abundance of microbial species, providing further support for the notion that gut community can have a profound effect on human gut function and dysfunction. Here, our analytical objectives were to (i) determine if the species-level differentiation between these healthy and IBD samples represents community-level divergence in the prevalence of metabolic networks, and (ii) gain insights into the metabolic basis of the differences between those gut environments. We processed the data to obtain EC assignments and reaction abundances (Text S5). Reactions were converted into substrate-product pairs and analyzed using BiomeNet. The processed data, formatted for input to BiomeNet are available with the source code at: http://sourceforge.net/projects/biomenet/. Reaction abundances, as input into BiomeNet, ranged from 5,091 to 374,945 counts per sample (see Text S5 for further detail about how reaction counts are obtained from raw reads). We initially evaluated *K* = 3 metabosystems based on categories for health status (healthy, Crohn's disease & ulcerative colitis). Recall that the model will not assign a health status to a particular metabosystem.

**Figure 6:**
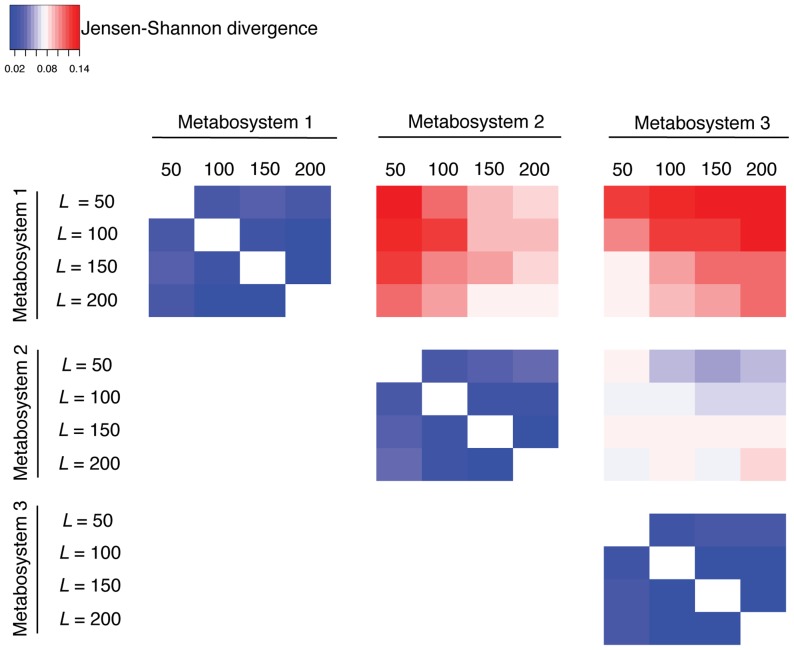
Divergence of reaction composition within and between the three metabosystems inferred from the gut-microbiomes of healthy humans and IBD patients. Divergence between metabosystems is measured at the level of their reaction composition by using the Jensen-Shannon divergence (JSD), and presented as a heat map. Further details about computing the reaction composition of a metabosystem are given in Text S3. The JSD score is used here to provide a symmetric measure of the difference between the composition of all 3,433 reactions in these data. The heat map is comprised of six divergence matrices, one for each of the possible pairwise comparisons between the metabosystems. The three matrices along the diagonal represent comparisons within the same metabosystem for different numbers of subnetworks in the model (*L* = 50, 100, 150 and 200); the dominance of blue (low JSD) in those matrices indicates that reaction composition is robust. The three off-diagonal matrices represent comparisons between metabosystems for different numbers of subnetworks (*L*); larger JSD scores here indicate greater divergence between metabosystems.

Again, we used JSD scores to measure divergence between metabosystems according to their reaction composition, and we visualized the divergence patterns by using a heat map (Figure 6). As with the mammal dataset above, the diagonal matrices (within metabosystem comparisons) are characterized by low JSD scores and the off-diagonal matrices (between metabosystem comparisons) are characterized by higher JSD scores. This confirms that characteristic reaction compositions can be identified when *K* = 3. Again, the three metabosystems are not equally divergent; while metabosystems 2 and 3 are divergent from each other, they are even more divergent from metabosystem 1. We also evaluated a model having *K* = 2 metabosystems, and found that no separation of samples was possible. Because each metabosystem has a characteristic reaction composition, it was easy to coordinate metabosystems across the different analyses when *K* = 3 (Text S6 and Figures 1and 2 in Text S6). Although *L* = 50 appears adequate for these data (Figure 6), for consistency we present the results derived from a model with *L* = 100 (and we provide the mixture probabilities of reactions in those subnetworks in Data File S1).

**Figure 7:**
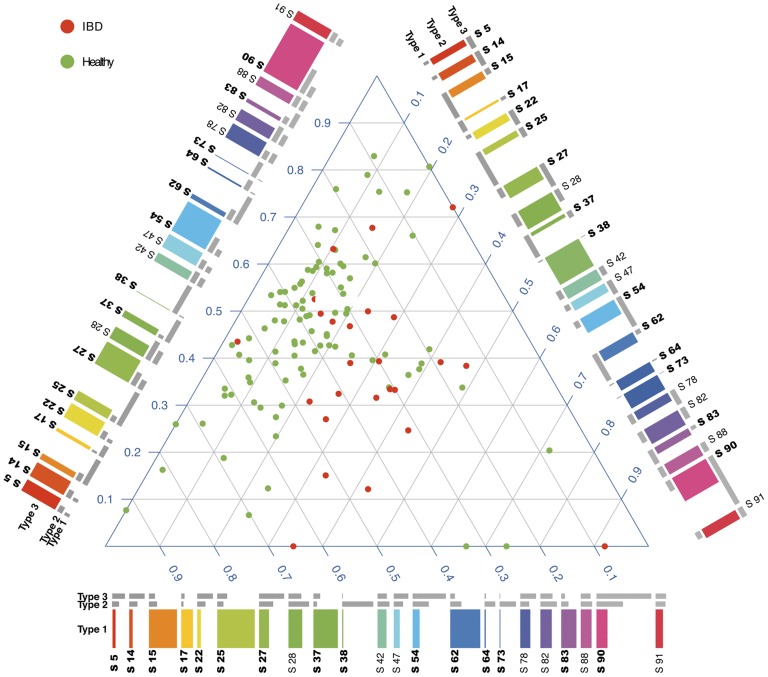
Inferred metabolic composition of the gut-microbiomes of healthy humans and IBD patients. Human microbiome samples are mapped to 3 metabosystems and plotted on a simplex (de Finetti diagram). Each point in the simplex is a different human sample. The metabosystem proportions for each sample sum to one. Lower left, lower right and top corners of the simplex plot indicate 100% membership to metabosystems 1, 2 and 3 respectively. Samples with IBD are represented as red dots and healthy samples are in green. Each metabosystem is represented in terms of 22 principal subnetworks by using a composition ribbon plotted along each side of the plot. Metabosystem 1 and its principal subnetworks are plotted along the horizontal side of the simplex plot. The colored bars represent the membership of the subnetworks corresponding to the selected metabosystem (*e.g*., metabosystem 1 is selected in the horizontal ribbon plot). The other two sets of grey bars represent the membership of the same subnetworks in the other two metabosystems for comparison. Bold labels indicate the discriminatory subnetworks. For example, subnetwork 25 is substantially more abundant in metabosystem 1 compared to the other two metabosystems. The criteria for selecting principal subnetworks and discriminatory subnetworks are provided in Text S3. The composition plot for all 100 subnetworks can be found as Text S8.

**Figure 8:**
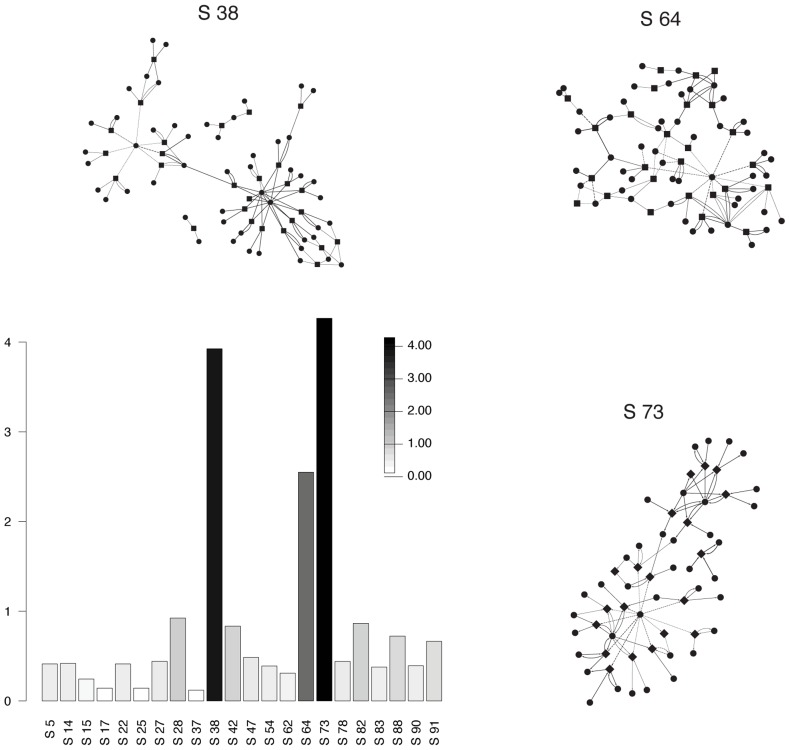
Discriminatory subnetworks for IBD patients. This histogram gives the relative membership score of subnetworks in metabosystem 2, which is found in higher proportion in IBD patients, compared to the other two metabosystems. The membership score for a subnetwork in a selected metabosystem is based on the ratio of its membership probability in this metabosystem to its largest membership probability in the other two metabosystems. The score is the absolute value of the logarithm of this ratio. Scores are plotted for principal subnetworks. Principal subnetworks for this dataset are defined as subnetworks with a membership greater than 2/*L* to at least one metabosystem. A similar plot for all subnetworks is provided as Text S8. Note that relative membership scores are computed solely for the purpose of visual assessment of metabosystem composition; the individual value of this score should not be attributed any additional meaning. It is easy to see that subnetworks 38, 64 and 73 are discriminatory for metabosystem 2. The discriminatory subnetworks also are depicted as networks. These networks illustrate a high degree of connectivity. Reactions are shown as square nodes and compounds are plotted as circular nodes. Links originating or ending in so-called currency compounds are represented with dashed lines.

Figure 7 presents the contribution of different metabosystems to each sample. Note that there are 41 obese individuals (body mass index>30) among the samples plotted in Figure 7, but we found no evidence for obesity-related metabolic systems. Neither could we separate the Crohn's and ulcerative colitis patients by using these data; however, only 4 of the 25 IBD samples were from Crohn's patients. Given that inferences must be made according to 3,433 reactions spread among 100 subnetworks, those 4 samples may have contained insufficient signal to characterize the Crohn's patients. Expanded sampling of Crohn's patients could be more informative. However, we did discover that the gut samples of IBD patients (red points) had a generally larger contribution from metabosystem 2, as compared to the healthy individuals (green points) who have a consistently low contribution (*θ_2_*<20%). We found that 15 of the 22 principal subnetworks (mixture weight>2/*L*) in these data make a large contribution to the divergence among metabosystems. Three of those 15 subnetworks (38, 64, and 73) are diagnostic of metabosystem 2 due to their very large relative contribution to that metabosystem (Figures 7 & 8 and Text S8). Reactions within those subnetworks are involved in amino sugar metabolism, ascorbate metabolism, fructose and mannose metabolism, aminobenzoate degradation, glycolysis and gluconeogenesis, as well as additional pathways (Tables S2, S3 & S4). Those subnetworks do not correspond to full KEGG pathways, or even contiguous subsets of those pathways. However, inspection of Figure 8 reveals that their reactions are indeed connected. Interestingly, reactions belonging to the non-discriminatory, or “core”, subnetworks do tend to comprise contiguous subsets of the main KEGG pathways (Text S9).

As the contribution of metabosystem 2 is elevated in IBD patients, we assessed the functional implications of their principal reactions (mixing probabilities>2/*R*) within subnetworks 38, 64 and 73. Subnetworks 38, 64 and 73 were comprised of 29, 19 and 18 principal reactions, respectively. Within each subnetwork, the principal reactions had a cumulative probability density>0.99 (Data File S1). The KEGG reaction numbers, reactant numbers and pathways, as well as mixing probabilities, are given for the principal reactions in Tables S2 to S4. Subnetworks 38, 64 and 73 contained reactions that are relevant to the phenotype of IBD.

One way to validate the IBD associated signal is to seek concordance with results obtained from independent data. Although there are no other metagenome datasets that are suitable for a similar investigation under BiomeNet, two different taxonomic studies [38], [39] attempted “indirect inference” of metabolic capacity in different sets of IBD samples. Those studies mapped 16S reads to the genomes of reference species, applied an algorithm to predict metagenome composition, and then used the metabolic capacity of the predict metagenomes to make inferences about the IBD phenotype. For certain metabolic systems, we observed a remarkable degree of concordance with thier findings. We found an enrichment of genes involved in the phosphotransferase (PTS) system (within subnetwork 38), and Morgan et al. [38] inferred that this system was more abundant in their IBD samples. We found an enrichment of genes associated with the ability to deal with oxidative stress (within subnetwork 64), and Morgan et al. [38] inferred a major shift in oxidative stress pathways with IBD. We found an enrichment of genes involved in the aminobenzoate degradation (within subnetwork 73), and Gevers et al. [39] inferred an association between this pathway and their IBD samples. The similarity of the IBD-related signal in these different datasets represents an important cross-validation for all the studies. At the individual reaction level, our results do differ somewhat from theirs; however this is expected, at least in part, because community metabolic function can only be indirectly assessed via 16S. Below we discuss in detail the key reactions that we have identified and, where possible, we derived hypotheses having explicitly testable predictions.

PTS reactions for various sugars (D-mannitiol, D-fructose, D-mannose) are abundant in subnetwork 38, and hence metabosystem 2. Reactions responsible for transport of N-acetylglucosamine and fucose are potentially meaningful because they suggest that metabosystem 2 is more dependent on glycans derived from host-associated substances, like mucin and shed epithelial cells, as a source of energy. Although these transporters do not point directly at the cause of gut inflammation associated with IBD, it does suggest that establishment of community metabosystem 2 could explain the resistance of IBD to moderate dietary intervention because the endogenous glycans provide a persistent source of nutrients for the community. Interestingly, extreme intervention whereby an exclusive liquid diet is administered up to 12 weeks (called EEN treatment) can sometimes induce remission of IBD in pediatric cases [40]. Taxonomic surveys confirm that EEN alters gut bacterial composition in those cases [41], [42], supporting the hypothesis that IBD may be associated with a dysfunctional community [43]. The hypothesis that IBD is associated with a community having the capacity of subnetwork 38 could be tested if future clinical investigation of EEN were to include sampling of the gut microbiome. The first prediction is that subnetwork 38 should be prevalent in the IBD samples prior to EEN treatment. Second, if remission is associated with displacing a dysfunctional community, then we predict that subnetwork 38 (and, more broadly, metabosystem 2) should exhibit a significant decline in those cases that responded to EEN.

Subnetwork 64 contains reactions associated with ascorbate metabolism. Two reactions that convert ascorbate to dehydro-gulonate-6P have high relative-abundance in IBD patients suggesting the potential for reduced ascorbate levels within the gut due to microbiome metabolic activity. Remarkably, direct measurement reveals reduced ascorbate levels in IBD patients [44]. Since ascorbate absorption in the gut depends, in part, on a localized concentration gradient, its decomposition by gut bacteria could interfere with the human cells capacity to absorb it. This represents a critical link between metabosystem 2 and IBD. The chronic intestinal pathophysiology of IBD patients is related to the increased production of reactive oxygen and nitrogen species, leading to oxidative stress within the intestinal mucosa [45]–[47]. IBD patients have reduced antioxidants levels within the intestinal mucosa [48], and the severity of the disease is correlated with antioxidant levels and oxidative stress markers [48]. Ascorbate is an antioxidant that is reduced within the inflamed tissues of IBD patients [44], and the association of metabosystem 2 with IBD suggest that this metabolic phenotype could be interfering with the human cells capacity to absorb ascorbate.

Subnetwork 73 is enriched in genes involved in the modification and transport of sugars and the metabolism of benzoate (compound C00180 in Table S4). The direct relationship of subnetwork 73 to the IBD phenotype is unclear. However, the reactions involving benzoate suggest that the microbiota in IBD patients might differ from healthy individuals in how they influence the metabolic processing of dietary aromatic compounds. Metabolism of such compounds typically occurs within the gut through several intermediates to benzoate, which can be subsequently converted to hippurate [49]. Williams et al. [50] found that urinary hippurate excretion was significantly reduced in IBD cohorts, and that it was not due to any intrinsic metabolic deficiencies, nor to diet. Indeed, this hippurate deficiency is so reliable that it is now considered an important criterion for diagnosing IBD via urinary metabolic profiling [51]. The critical role of the microbiota in the metabolism of dietary aromatic compounds is supported by experimentation on mice and rats. Germ free animals do not excrete hippurate, but it comes to dominate 2–3 weeks after exposure to environmental microbes [52]. Conversely, treatment with antibiotics eliminates the production of hippurate in mice and rats [53]. It is interesting that the enzyme hippurate hydrolase is also enriched in subnetwork 73. Given a possible connection to IBD, the reactions of subnetwork 73 are good candidates for further study.

Bacteria inhabiting the luminal side of the mucosa are thought to play key role in human intestinal health and disease [*e.g*., 27; 54; 55]. Although colonic mucus is an effective barrier against infection of the epithelium, bacteria do colonize and persist within the outer mucosal layer, and explicitly utilize it as an energy source [27], [43]. The colon is characterized by invaginations within the epithelium (called enfolding crypts) that are laden with mucosal gel [56]. Recent work combining culture-independent qPCR and laser capture microdissection confirmed that the mucos gel within the crypts represent an exploitable niche in both healthy and diseased hosts [57]. Further, 16S-based comparison of mucosal biopsies and faecal samples indicate that their community composition is different [58]. As this is such a close association, the varied effects of the mucosal bacteria are believed to be elicited via the by-products of their metabolism [results from experimental models reviewed in 43]. Interestingly, individual bacteria do not appear to possess the enzymes necessary to cleave all mucin linkages; thus, the ability to colonize and persist within the mucosal gel appears to require community metabolic activities [43]. The capacity of community metabosystem 2 to exploit host-derived glycans suggests a close association with the host mucosa. If this notion is correct, then the ascorbate metabolic phenotype of this community could impact on the state of human health. Unfortunately working with stool samples limits our ability to attribute results to the colonic mucosal community, as such samples are comprised of a mixture of luminal and colonic mucosal communities [27], [58]. However, our results do lead to a testable hypothesis; if community metabosystems 2 is associated with the mucosal gel, then there should be a higher prevalence of metabosystem 2 in mucosal biopsy samples, as compared to stool samples, taken from the same individual having IBD.

Because members of the gut bacterial community provide real benefits to the host (digestion and metabolism, resistance of pathogen colonization, immune maturation [4], [5], [59]), selection of beneficial species by the host (partner selection) is often assumed to be an important part of the host-microbiome interaction. Recent work modeling microbial bioconversion [60] illustrated how host secretion of nutrients at the epithelium-microbiota interface (in addition to antimicrobial factors) could act as a powerful mechanism for partner selectivity [60]. The models also reveal that in the absence of nutrient-based partner selection, the slower growing strains will be lost, along with any beneficial effects they might provide to the host [60]. Interestingly, mucosal glycans secreted by the host are known to impact the attachment and growth of bacteria within the gut [61]. Thus the increased prevalence of community metabosystem 2 in IBD samples could be due to the growth of opportunistic bacteria on the epithelium that can utilize, or even circumvent, the host's nutrient-based mechanisms for partner selection. Intriguing work in a mouse model support the notion that IBD might be related to failure of an alternative mechanism of partner selection [62]. Garret et al. [62] showed that mice defective in T-bet (a transcription factor that regulates immune system cells) will spontaneously develop ulcerative colitis. The disease state in these mice is due to a community, rather than a single transmissible agent. Although this community does not arise spontaneously in wild type mice, the colitis phenotype is inducible in wild type mice via the “transmission” of the community [62]. T-bet represents another potential mechanism of partner selection. The challenge will be to understand how partner selection might be weakened within the human gut, and if this can lead to the establishment of communities with metabolic interactions like those of metabosystem 2.

## Discussion

We presented BiomeNet, a Bayesian mixture model that uses metagenome data to learn the structural features of microbial community metabolism. The model learns how reactions combine to form subnetworks, and how those subnetworks combine to form metabosystems. Although there are several issues that deserve further consideration and development (discussed below), our analyses with the current implementation were informative. For example, we found that samples of IBD patients have a high prevalence of a community metabosystem that we hypothesize is resistant to moderate dietary intervention, is closely associated with the human gut epithelium, and reduces the availability of an important antioxidant. Our finding that reactions within the most discriminatory subnetworks, although being highly connected, do not map to contiguous subsets of the main KEGG pathways illustrates the importance of learning the structures of the networks from the data.

A wide variety of problems in computational biology have been addressed by using probabilistic graphical models [63], including the problem of modeling pairwise relational data derived from a network [e.g., 64-66]. The mixed membership stochastic block model (MMSB) [65] is perhaps the most similar to BiomeNet, as it is a generative model for groups (such as communities) within a network. Under the MMSB model, the block structure is employed to model connectivity at the group level, and the mixed membership structure allows nodes to belong to multiple groups. BiomeNet is fundamentally different from MMSB in two ways. First, the MMSB model assumes that a network exists as a single entity, and the task is to model just a single realization of the network. BiomeNet treats the observed relational data as constrained by some underlying network structure, and the relational data represent many different realizations of the network constraints over samples. Thus we model the realization of links constrained within a network structure, whereas MMSB models the existence of links (or the weights on links) between nodes of one “snapshot” of a network. Second, MMSB and similar models are used to group nodes based on their links with other nodes in the network. BiomeNet is used to decompose a network into overlapping subnetworks. Because our pairwise relations are derived from known transformations between substrates and products, the subnetworks in BiomeNet have a direct functional interpretation. In addition to these conceptual differences, BiomeNet is structurally different from MMSB. We have a nested hierarchy of groupings whereas the MMSB model has only one level of grouping. The hierarchical structure of BiomeNet permits sharing of subnetworks across multiple networks, and this allows for a natural way of grouping multiple networks. The MMSB model only allows grouping of nodes within a single network. BiomeNet is also better suited for networks containing hyper-edges because it preserves some of the interdependencies by conditioning pairs of edges on the subnetwork assignments; the other approaches [64]–[66] break hyper-edges into independent pairwise relations.

To validate the BiomeNet inference algorithm, we simulated hierarchical network data over many different combinations for the number of compounds, reactions, subnetworks and metabosystems. Reliable estimates of the mixing probabilities under this design, even when there was a large discrepancy between the fixed value of the concentration parameter and the true value for the generating process, confirmed the reliability of the algorithm. We note, however, that there are other steps involved in the processing of real metagenomic samples that are not included in our generative process (*e.g*., scaling counts, mapping reads to EC numbers, etc.). Decisions must be made during the processing of any metagenomic dataset, and these could impact downstream analysis of community level metabolic capacity. Clearly, further simulation-based research is needed to investigate the sensitivity of various analytical methods (including BiomeNet) to the upstream details of data collection and processing. Such a comprehensive investigation is beyond the scope of this study.

A common criticism of Bayesian methods relates to the need to specify a prior probability distribution for every unknown parameter in the model, even when prior knowledge about the parameter is vague or incomplete. In BiomeNet the prior distributions are controlled by concentration parameters of the Dirichlet distribution. We were not concerned with constructing a so-called “objective” Bayesian method [67], where unbiased knowledge of the biological process is used to correctly describe the uncertainties in the model parameter (or, where lacking such information a non-informative prior is employed), nor did we desire a prior that was necessarily complete in its ability to describe our personal degree of belief about alternative values for the parameters of BiomeNet. Rather, we followed Gelman and Shalizi [30] by viewing the prior as serving several functions, and premier among these was setting the concentration parameters to promote our desire for certain properties (reduced variance and improved identifiably). Specifically, we set the concentration parameters close to zero, which encourages BiomeNet to predominantly characterize metabosystems by a relatively few major subnetworks, and subnetworks by a relatively few major reactions. This aids the interpretability of results. An alternative approach would be to place second-stage priors (hyper-priors) on the concentration parameters of the Dirichlet distribution, thereby allowing for uncertainties in the concentration parameters by automatically including them in the posterior distribution [68]. This is an interesting area for future development of BiomeNet, as it would allow estimation of the priors, through the hyper-priors, from the data.

We chose to use the terms “subnetwork” and “metabosystem”, rather than “pathway” and “community”, so as to clearly delineate our model-derived structures from those entities having more biology-centred definitions (*e.g*., a human-curated metabolic pathway or an ecological community). Indeed, microorganisms within a microbiome sample are expected to show metabolic interactions that range from negligible to obligate. We followed Boon et al. [69] in reserving the term “community” for those sets of microorganisms having a high degree of ecological interaction. Thus the metabosystems that are detected by using BiomeNet can be considered the starting points for detecting metabolically integrated communities. The notion of metabolic integration at the community level would entail experimental validation [69]. Metabosystems could be directly tested for ecological stability by experimentally disturbing a microbiota (*e.g*., via antibiotic treatment) and assessing the extent to which that system returns to the same pre-disturbance composition of subnetworks.

It is important to note that we are working with a microbiome's latent capacity to process metabolites, as inferred from its metagenome. Thus the structures referred to as “metabosystems” reflect a latent aspect of metabolic phenotype. We modelled each sample as a mixture of metabosystems because microbial communities are thought to be composed of partially overlapping assemblages of microbes with varying types of ecological interaction [70], [71]. By modelling a sample as a mixture of metabolic structures, we must also adopt a “softened” (probabilistic) definition of metabolic phenotype. We believe this will ultimately benefit our understanding of how the observed variability in community function might relate to phenotypic labels (*e.g*., “diseased” or “healthy”). Our modelling approach is therefore suited to investigating questions about how the level of an individual's health status might be determined by mixtures of different metabolic phenotypes.

BiomeNet provides the capability to focus attention on the fundamental structure of a complex metabolic network, which is necessary if researchers want to improve their understating of microbial community function. We note that this will also be the case when seeking a better understanding of the taxonomic composition of community structures. Along these lines, Arumugam and coauthors [72] suggested that community structure within the human gut can be adequately characterized in terms of three distinct community types, called “enterotypes”. However, this suggestion has generated some controversy [73]. Other researches suggested that human gut microbiome might be better described by two distinct enterotypes [74], although the inferred number depends to some degree on methodology [75]. More importantly, there is no consensus on how to define an enterotype, nor a consensus on what biological significance, if any, should be attributed to the inferred number, because the underlying diversity of the human gut seems to exist as a continuum of communities [75]–[77]. The challenge, as we see it, is the need to formally model community structures (or functions, as in the case of BiomeNet) as entities having “soft boundaries” so that samples can be more realistically characterized as mixtures of communities. There has been only limited development of this approach for taxonomic composition of communities [78]–[80]. The composition models [78]–[80] are similar to BiomeNet in applying a Dirichlet prior to the parameters of the multinomial. However, because their objective is to model structure in terms of 16S based phylotypes, they do not model community structure as an explicit network as is done by BiomeNet. Furthermore, only one composition model is hierarchical [80]. The hierarchical model [80] attempts to capture the most realistic mixture of community structures; its goal is to learn how phylotypes are mixed to form assemblages, how assemblages are mixed to form community samples, and how community structure is related to phenotypes of interest. We suggest that using a “soft” (probabilistic) definition of community structure (whether taxonomic or function-based) rather than a “hard” (discrete) definition better serves the goal of microbiome research to discover the different aspects of microbial communities and link them to the relevant phenotypes.

BiomeNet is suitable to investigating the latent metabolic structure of any microbial community. As long as abundance data can be obtained for sequences with assigned metabolic function, the model can be applied to samples from any environment (soil, open ocean, biofilm reactors, etc.) and over any scale (distance, time, etc.). BiomeNet can easily accommodate the abundance data generated from high-throughput sequencing of RNA transcripts of uncultivable microorganisms. Thus, it could be employed to investigate the temporal dynamics of community metabolic structure within serially-sampled metatranscriptomic data.

## Supporting Information

Text S1 **Network generation under the model, and validation of the inference algorithm.** An overview of the generative process under BiomeNet and a detailed description of how we used simulation to verify that our sampling algorithm can recover the parameter values used to generate reactions for simulated microbiome samples.
(PDF)

Text S2 **Analytical solutions for the individual terms of the posterior distribution of the latent variables.** For inference under BiomeNet we sample from the posterior distribution of latent variables given the data. We use collapsed Gibbs sampling by integrating out the latent variables *θ, φ, δ, γ* and sample from the posterior distributions of the metabosystem (*Z*) and subnetwork (*Y*) assignments for each reaction conditional on the assignments of all other reactions. This is a high dimensional distribution, and the analytical solutions for the individual terms are given in these notes.
(PDF)

Text S3 **Characterizing the reaction composition of a metabosystem, and measuring compositional differences between metabosystems.** A detailed description of the method for characterizing the composition of a metabosystem at the level of its metabolic reactions, the method for measuring compositional differences between metabosystems, and the method for assessing robustness of metabosystems to the *L* value. The criteria for classifying principal subnetworks and discriminatory subnetworks are also provided.
(PDF)

Text S4 **PCoA analysis of mammalian microbiome samples.** PCoA analysis of Enzyme Commission assignments and KEGG Orthology assignments for the mammalian dataset reveals a separation between carnivore and herbivore gut microbiomes.
(PDF)

Text S5 **Source and processing of metagenomic data.** The source of the two gut metagenome datasets and a description how those data were processed to obtain abundance values for substrate-product pairs that were input into the model.
(PDF)

Text S6 **Robustness of metabosystem composition to number of subnetworks.** Evaluation of alternative numbers of subnetworks (*L*) in the model reveals that the reaction composition of the metabosystems is relatively stable to the specified value of *L*.
(PDF)

Text S7 **Subnetwork composition of mammalian metabosystems.** A presentation of the inferred contribution of 100 subnetworks to three metabosystems in the model, and the identification of the most discriminatory subnetwork for metabosystem 1.
(PDF)

Text S8 **Subnetwork composition of the human IBD/healthy metabosystems.** A presentation of the inferred contribution of 100 subnetworks to three metabosystems in the model, and the identification of the most discriminatory subnetworks for the metabosystem that was most associated with IBD (metabosystem 2).
(PDF)

Text S9 **Core subnetworks.** Definition and identification of core subnetworks under the BiomeNet modeling framework.
(PDF)

Table S1 **Composition of subnetwork 49 inferred from the mammalian dataset.** The table gives the KEGG reaction numbers, substrates and products for the principal reactions in mammalian subnetwork 49. Because the model does not rigidly define subnetworks, each reaction in the dataset will have an estimated mixing probability. As the majority of reactions make only a trivial contribution to this subnetwork (nearly zero), we filtered out any reaction with a contribution less than 2/*R*, where *R* is the count of unique reactions summed over all the samples in a dataset. This resulted in a subset of 25 reactions having a posterior density>0.99.
(PDF)

Table S2 **Composition of subnetwork 38 inferred from the human dataset.** The table gives the KEGG reaction numbers, substrates and products for the principal reactions in human subnetwork 38. Because the model does not rigidly define subnetworks, each reaction in the dataset will have an estimated mixing probability. As the majority of reactions make only a trivial contribution to this subnetwork (nearly zero), we filtered out any reaction with a contribution less than 2/*R*, where *R* is the count of unique reactions summed over all the samples in a dataset. This resulted in a subset of 29 reactions having a posterior density>0.99.
(PDF)

Table S3 **Composition of subnetwork 64 inferred from the human dataset.** The table gives the KEGG reaction numbers, substrates and products for the principal reactions in human subnetwork 64. Because the model does not rigidly define subnetworks, each reaction in the dataset will have an estimated mixing probability. As the majority of reactions make only a trivial contribution to this subnetwork (nearly zero), we filtered out any reaction with a contribution less than 2/*R*, where *R* is the count of unique reactions summed over all the samples in a dataset. This resulted in a subset of 19 reactions having a posterior density>0.99.
(PDF)

Table S4 **Composition of subnetwork 73 inferred from the human dataset.** The table gives the KEGG reaction numbers, substrates and products for the principal reactions in human subnetwork 73. Because the model does not rigidly define subnetworks, each reaction in the dataset will have an estimated mixing probability. As the majority of reactions make only a trivial contribution to this subnetwork (nearly zero), we filtered out any reaction with a contribution less than 2/*R*, where *R* is the count of unique reactions summed over all the samples in a dataset. This resulted in a subset of 18 reactions having a posterior density>0.99.
(PDF)

Data File S1 **Posterior mixing probabilities of the principal reactions in all subnetworks (** ***L*** **= 100).** Posterior probabilities are given for both the mammalian and human datasets. Because many reactions will have trivially small mixing probabilities for many subnetworks (nearly zero), we filtered out any reaction with a contribution less than 2/*R*, where *R* is the count of unique reactions summed over all the samples within the given dataset. The data are presented in a spreadsheet document, with the mammalian dataset and the human IBD/healthy dataset provided within separate sheets. Each sheet has 100 rows, one for each subnetwork in the model. Each row provides the KEGG reaction ID and mixing probabilities for a particular subnetwork. The data in each row is sorted according to its mixing probabilities.
(XLSX)

## References

[1] Savage DC (1977) Microbial ecology of the gastrointestinal tract. Annu Rev Microbiol 31: 107–133. 334036 10.1146/annurev.mi.31.100177.000543

[2] Phelan VV, Liu WT, Pogliano K, Dorrestein P (2012) Microbial metabolic exchange-the chemotype-to-phenotype link. Nat Chem Biol 8: 26–35. 22173357 10.1038/nchembio.739 PMC3869239

[3] Arrigo KR (2005) Marine microorganism and global nutrient cycles. Nature 437: 349–355. 16163345 10.1038/nature04159

[4] Fujimura K, Slusher N, Cabana M, Lynch S (2010) Role of the gut microbiota in defining human health. Expert Rev Anti Infect Ther 8: 435–454. 20377338 10.1586/eri.10.14 PMC2881665

[5] Sekirov I, Russell S, Antunes L, Finleay B (2010) Gut microbiota in health and disease. Physiol Rev 90: 859–904. 20664075 10.1152/physrev.00045.2009

[6] Handelsman J (2004) Metagenomics: Application of genomics to uncultured microorganisms. Microbiol Mol Biol Rev 68: 669–685. 15590779 10.1128/MMBR.68.4.669-685.2004 PMC539003

[7] Riesenfeld CS, Schloss PD, Handelsman J (2004) Metagenomics: Genomic analysis of microbial communities. Annu Rev Genet 38: 525–552. 15568985 10.1146/annurev.genet.38.072902.091216

[8] Schloss PD, Handelsman J (2005) Metagenomics for studying unculturable microorganisms: cutting the Gordian knot. Genome Biol 6: 229. 16086859 10.1186/gb-2005-6-8-229 PMC1273625

[9] Earth Microbiome Project (2012)Available at www.earthmicrobiome.org

[10] Peterson J, et al (2009) The NIH Human Microbiome Project. Genome Res 19: 2317–2323. 19819907 10.1101/gr.096651.109 PMC2792171

[11] Kuczynski J, et al (2012) Experimental and analytical tools for studying the human microbiome. Nat Rev Genet 13: 47–58. 22179717 10.1038/nrg3129 PMC5119550

[12] Dinsdale EA, Edwards RA, Hall D, Angly F, Breitbart M, et al (2008) Functional metagenomic profiling of nine biomes. Nature 452: 629–632. 18337718 10.1038/nature06810

[13] Muegge B, Kuczynski J, Knights D, Clemente JC, González A, et al (2011) Diet drives convergence in gut microbiome functions across mammalian phylogeny and within humans. Science 332: 970–973. 21596990 10.1126/science.1198719 PMC3303602

[14] Gianoulis TA, Raes J, Patel PV, Bjornson R, Korbel JO, et al (2009) Quantifying environmental adaptation of metabolic pathways in metagenomics. Proc Natl Acad Sci U S A 106: 1374–1379. 19164758 10.1073/pnas.0808022106 PMC2629784

[15] Ye Y, Doak TG (2009) A parsimony approach to biological pathway reconstruction/inference for genomes and metagenomes. PLoS Comput Biol 5: e1000465. 19680427 10.1371/journal.pcbi.1000465 PMC2714467

[16] Liu B, Pop M (2011) MetaPath: identifying differentially abundant metabolic pathways in metagenomic datasets. BMC Proc (Suppl 2): S9. 10.1186/1753-6561-5-S2-S9 PMC3090767 21554767

[17] Abubucker S, Segata N, Goll J, Schubert AM, Izard J, et al (2012) Metabolic reconstruction for metagenomic data and its application to the human microbiome. PLoS Comput Biol 8: e1002358. 22719234 10.1371/journal.pcbi.1002358 PMC3374609

[18] Jiao D, Ye Y, Tang H (2013) Probabilistic inference of biochemical reactions in microbial communities from metagenomic sequences. PLoS Comput Biol 9: e1002981. 23555216 10.1371/journal.pcbi.1002981 PMC3605055

[19] Meyer F, Paarmann D, D'Souza M, Olson R, Glass EM, et al (2008) The metagenomics RAST server: a public resource for the automatic phylogenetic and functional analysis of metagenomes. BMC Bioinformatics 9: 386. 18803844 10.1186/1471-2105-9-386 PMC2563014

[20] Qin J, Li R, Raes J, Arumugam M, Burgdorf KS, et al (2010) A human gut microbial gene catalogue established by metagenomic sequencing. Nature 464: 59–65. 20203603 10.1038/nature08821 PMC3779803

[21] Chipman H, Gu H (2005) Interpretable Dimension Reduction. J Appl Stat 32: 969–987.

[22] Larsen PE, Collart FR, Field D, Meyer F, Keegan KP, et al (2011) Predicted Relative Metabolomic Turnover (PRMT): determining metabolic turnover from a coastal marine metagenomic dataset. Microb Inform and Exp 1: 4. 10.1186/2042-5783-1-4 PMC3348665 22587810

[23] Kanehisa M (2013) Chemical and genomic evolution of enzyme-catalyzed reaction networks. FEBS Lett 587: 2731–2737. 23816707 10.1016/j.febslet.2013.06.026

[24] Ravasz E, Somera AL, Mongru DA, Oltvai ZN, Barabási AL (2002) Hierarchical organization of modularity in metabolic networks. Science 297: 1551–1555. 12202830 10.1126/science.1073374

[25] Kreimer A, Borenstein E, Gophna U, Ruppin E (2008) The evolution of modularity in bacterial metabolic networks. Proc Natl Acad Sci USA 105: 6976–6981. 18460604 10.1073/pnas.0712149105 PMC2383979

[26] Morine MJ, Gu H, Myers RA, Bielawski JP (2009) Trade-offs between efficiency and robustness in bacterial metabolic networks are associated with niche breadth. JMol Evol 68: 506–515. 19365645 10.1007/s00239-009-9226-5

[27] Koropatkin NM, Cameron EA, Martens EC (2012) How glycan metabolism shapes the human gut microbiota. Nat Rev Microbiol 10: 323–335. 22491358 10.1038/nrmicro2746 PMC4005082

[28] Casella G, George EI (1992) Explaining the Gibbs sampler. Am Stat 46(3): 167–174.

[29] Liu JS (1994) The collapsed Gibbs sampler in Bayesian computations with applications to a gene regulation problem. J Am Stat Assoc 89: 958–966.

[30] Gelman A, Shalizi CR (2013) Philosophy and the practice of Bayesian statistics. Br J Math Stat Psychol 66: 8–38. 22364575 10.1111/j.2044-8317.2011.02037.x PMC4476974

[31] Hastie T, Tibshiriani R, Freidman J (2001) The elements of statistical learning; data mining, inference, and prediction. Springer series in statistics. Springer, New York 552 p.

[32] Lin J (1991) Divergence measures based on the Shannon Entropy. IEEE Trans Inf Theory 37: 145–151.

[33] Ghuysen J (1968) Use of bacteriolytic enzymes in determination of wall structure and their role in cell metabolism. Bacteriol Rev 32: 425–464. 4884715 PMC413160

[34] Ghuysen JM, Strominger JL (1963) Structure of the cell wall of Staphylococcus aureus, strain Copenhagen. II. Separation and structure of disaccharides. Biochemistry 2: 1119–1125. 14087370 10.1021/bi00905a036

[35] Macfarlane S, Woodmansey E, Macfarlane G (2005) Coloniziation of mucin by human intestinal bacteria and establishment of biofilm communities in a two-stage continuous culture system. Appl Environ Microb 71: 7483–7492. 10.1128/AEM.71.11.7483-7492.2005 PMC1287682 16269790

[36] Sonnenburg J, Xu J, Leip DD, Chen CH, Westover BP, et al (2005) Glycan foraging in vivo by an intestine-adapted bacterial symbiont. Science 307: 1955–1959. 15790854 10.1126/science.1109051

[37] Hooper L, Zu J, Falk P, Midtvedt T, Gordon J (1999) A molecular sensor that allows a gut commensal to control its nutrient foundation in a competitive ecosystem. Proc Natl Acad Sci USA 96: 9833–9838. 10449780 10.1073/pnas.96.17.9833 PMC22296

[38] Morgan XC, Tickle TL, Sokol H, Gevers D, Devaney KL, et al (2012) Dysfunction of the intestinal microbiome in inflammatory bowel disease and treatment. Genome Biol 13: R79. 23013615 10.1186/gb-2012-13-9-r79 PMC3506950

[39] Gevers D, Kugathasan S, Denson LA, Vázquez-Baeza Y, Van Treuren W, et al (2014) The treatment-naïve microbiome in new-onset Crohn's disease. Cell Host Microbe 15: 382–392. 24629344 10.1016/j.chom.2014.02.005 PMC4059512

[40] Nahidi L, Day AS, Lemberg DA, Leach ST (2014) Paediatric inflammatory bowel disease: a mechanistic approach to investigate exclusive enteral nutrition treatment. Scientifica 2014: 423817. 24967146 10.1155/2014/423817 PMC4055462

[41] Pryce-Millar E, Murch SH, Heuschkel RB, Afzal N, Rampton DS, et al (2004) P0610 Enteral nutrition therapy in Crohn's disease changes the mucosal flora. J Pediatr Gasteroenterol Nutr 39: s289.

[42] Lionetti P, Callegari ML, Ferrari S, Cavicchi MC, Pozzi E, et al.. (2005) Enteral nutrition and microflora in pediatric Crohn's disease. JPEN J Parenter Enteral Nutr 29(4 Suppl): S173–S175. 10.1177/01486071050290S4S173 15980280

[43] Pearson JP, Brownlee IA (2010) The interaction of large bowel microflora with the colonic mucus barrier. Int J Inflam 2010: 321426.. 21152122 10.4061/2010/321426 PMC2989700

[44] Buffington G, Doe W (1995) Altered ascorbic acid status in the mucosa from inflammatory bowel disease patients. Free Radic Res 22: 131–143. 7704184 10.3109/10715769509147535

[45] Pavlick KP, Laroux FS, Fuseler J, Wolf RE, Gray L, et al (2002) Role of reactive metabolites of oxygen and nitrogen in inflammatory bowel disease. Free Radic Biol Med 33: 311–322. 12126753 10.1016/s0891-5849(02)00853-5

[46] Pravda J (2005) Radical induction theory of ulcerative colitis. World J Gastroenterol 11: 2371–2384. 15832404 10.3748/wjg.v11.i16.2371 PMC4305621

[47] Karp S, Koch T (2006) Oxidative stress and antioxidants in inflammatory bowel disease. Dis Mon 52: 199–207. 16828361 10.1016/j.disamonth.2006.05.005

[48] Zhu H, Li R (2012) Oxidative stress and redox signaling mechanisms of inflammatory bowel disease: updated experimental and clinical evidence. Exp Biol Med 237(5): 474–480. 10.1258/ebm.2011.011358 22442342

[49] Lees HJ, Swan JR, Wilson ID, Nicholson JK, Holmes E (2013) Hippurate: The natural history of a mammalian-microbial cometabolite. J Proteome Res 12: 1527–1546. 23342949 10.1021/pr300900b

[50] Williams HRT, Cox IJ, Walker DG, Cobbold JFL, Taylor-Robinson SD, et al (2010) Differences in gut microbial metabolism are responsible for reduced hippurate synthesis in Crohn's disease. BMC Gastroenterol 10: 108. 20849615 10.1186/1471-230X-10-108 PMC2954941

[51] De Preter V, Verbeke K (2013) Metabolomics as a diagnostic tool in gastroenterology. World J Gastrointest Pharmacol Ther 4: 97–107. 24199025 10.4292/wjgpt.v4.i4.97 PMC3817290

[52] Claus SP, Tsang TM, Wang Y, Cloarec O, Skordi E, et al (2008) Systemic multicompartmental effects of the gut microbiome on mouse metabolic phenotypes. Mol Syst Biol 4: 219. 18854818 10.1038/msb.2008.56 PMC2583082

[53] Yap IKS, Li JV, Saric J, Martin FP, Davies H, et al (2008) Metabonomic and microbiological analysis of the dynamic effect of vancomycin-induced gut microbiota modification in mice. J Proteome Res 7: 3718–3728. 18698804 10.1021/pr700864x

[54] Hughes R, Rowland IR. (2000) Metabolic activities of the gut microflora in relation to cancer. Microb Ecol Health Dis (Suppl 2): 179–185.

[55] Abreu MT (2010) Toll-like receptor signalling in the intestinal epithelium: how bacterial recognition shapes intestinal function. Nat Rev Immunol 10: 131–144. 20098461 10.1038/nri2707

[56] Gibson PR, Anderson RP, Mariadason JM, Wilson AJ (1996) Protective role of the epithelium of the small intestine and colon. Inflamm Bowel Dis 2(4): 279–302. 23282597

[57] Rowan F, Docherty NG, Murphy M, Murphy TB, Coffey JC, et al (2010) Bacterial colonization of colonic crypt mucous gel and disease activity in ulcerative colitis. Ann Surg 252(5): 869–875. 21037444 10.1097/SLA.0b013e3181fdc54c

[58] Eckburg PB, Bik EM, Bernstein CN, Purdom E, Dethlefsen L, et al (2005) Diversity of the human intestinal microbial flora. Science 308: 1635–1638. 15831718 10.1126/science.1110591 PMC1395357

[59] Hooper LV, Macpherson AG (2010) Immune adaptations that maintain homeostasis with the intestinal microbiota. Nat Rev Immunol 10: 159–169. 20182457 10.1038/nri2710

[60] Schluter J, Foster KR (2012) The evolution of mutalism in gut microbiota via host epithelial selection. PLoS Biol 10(11): e1001424.. 23185130 10.1371/journal.pbio.1001424 PMC3502499

[61] Hooper LC, Gordon JI (2001) Glycans as legislators of host-microbioal interactions: spanning the spectrum from symbiosis to pathogenicity. Glycobiology 11: 1R–10R. 11287395 10.1093/glycob/11.2.1r

[62] Garret WS, Lord GM, Punit S, Lugo-Villarino G, Mazmanian S, et al (2007) Communicable ulcerative colitis induced by T-bet deficiency in the innate immune system. Cell 131(1): 33–45. 17923086 10.1016/j.cell.2007.08.017 PMC2169385

[63] Airoldi EM (2007) Getting Started in Probabilistic Graphical Models. PLoS Comput Biol 3(12): e252. 18069887 10.1371/journal.pcbi.0030252 PMC2134967

[64] Newman MEJ, Leicht EA (2007) Mixture models and exploratory analysis in networks. Proc Natl Acad Sci U S A 104(23): 9564–9569. 17525150 10.1073/pnas.0610537104 PMC1887592

[65] Airoldi EM, Blei DM, Fienberg SE, Xing EP (2008) Mixed membership stochastic block models. J Mach Learn Res 9: 1981–2014. 21701698 PMC3119541

[66] Karrer B, Newman MEJ (2011) Stochastic blockmodels and community structure in networks. Phys Rev E 83: 016107. 10.1103/PhysRevE.83.016107 21405744

[67] Berger J (2006) The case for objective Bayesain analysis. Bayesian Anal 1: 385–402.

[68] Gelman A, Carlin JB, Stern HS, Rubin DB (1995). Bayesian Data Analysis. Chapman and Hall, London 696 p.

[69] Boon E, Meehan CJ, Whidden C, Wong DH, Langille MG, Beiko RG (2014) Interactions in the microbiome: communities of organisms and communities of genes. FEMS Microbiol Rev 38: 90–118. 23909933 10.1111/1574-6976.12035 PMC4298764

[70] Burke C, Steinberg P, Rusch D, Kjelleberg S, Thomas T (2011) Bacterial community assembly based on functional genes rather than species. Proc Natl Acad Sci USA 108: 14288–14293. 21825123 10.1073/pnas.1101591108 PMC3161577

[71] Patel PV, Gianoulis TA, Bjornson RD, Yip KY, Engelman DM, Gerstein MB (2010) Analysis of membrane proteins in metagenomics: networks of correlated environmental features and protein families. Genome Res 20: 960–971. 20430783 10.1101/gr.102814.109 PMC2892097

[72] Arumugam M, Raes J, Pelletier E, Le Paslier D, Yamada T, et al (2011) Enterotypes of the human gut microbiome. Nature 473: 174–180. 21508958 10.1038/nature09944 PMC3728647

[73] Yong E (2012) Gut Micorbial ‘enterotypes’ become less clear-cut. *NatureNews* [online] http://www.nature.com/news (12 Mar 2012)

[74] Wu GD, Chen J, Hoffmann C, Bittinger K, Chen YY, et al (2011) Linking long-term dietary patterns with gut microbial enterotypes. Science 334: 105–108. 21885731 10.1126/science.1208344 PMC3368382

[75] Koren O, Knights D, Gonzalez A, Waldron L, Segata N, et al (2013) A guide to enterotypes across the human body: meta-analysis of microbial community structures in human microbiome datasets. PLoS Comput Biol 9(1): e1002863. 23326225 10.1371/journal.pcbi.1002863 PMC3542080

[76] Huse SM, Ye Y, Zhou Y, Fodor AA (2012) A core human microbiome as viewed through 16S rRNA sequence clusters. PLoS One 7: e34242. 22719824 10.1371/journal.pone.0034242 PMC3374614

[77] Jeffery IB, Claesson MJ, O'Toole PW, Shanahan F (2012) Categorization of the gut microbiota: enterotypes or gradients? Nat Rev Microbiol 10: 591–592. 23066529 10.1038/nrmicro2859

[78] Knights D, Kuczynski J, Charlson ES, Zaneveld J, Mozer MC, et al (2011) Bayesian community-wide culture-independent microbial source tracking. Nat Methods 8: 761–763. 21765408 10.1038/nmeth.1650 PMC3791591

[79] Holmes I, Harris K, Quince C (2012) Dirichlet multinomial mixtures: generative models for microbial metagenomics. PLoS One 7: e30126. 22319561 10.1371/journal.pone.0030126 PMC3272020

[80] Shafiei M, Dunn KA, Boon E, MacDonald SM, Walsh DA, et al (Submitted) BioMiCo: a supervised Bayesian model for inference of microbial community structure. Microbiome 10.1186/s40168-015-0073-x PMC4359585 25774293

