# Supplementary material for: BiomeNet: A Bayesian Model for Inference of Metabolic Divergence among Microbial Communities
Source: PLoS Comput Biol. 2014 Nov 20;10(11):e1003918. doi: 10.1371/journal.pcbi.1003918 (PMC4238953; doi:10.1371/journal.pcbi.1003918)
Supplement: Text S1 — Network generation under the model, and validation of the inference algorithm. An overview of the generative process under BiomeNet and a detailed description of how we used simulation to verify that our sampling algorithm can recover the parameter values used to generate reactions for simulated microbiome samples. (PDF) [file pcbi.1003918.s001.pdf]

Text S1: Network generation under the model and validation of inference algorithm.

Our model assumes a process by which the data is generated. Therefore, it is straightforward to simulate data from the model. We provide an overview of the generative process, which we use to simulate data for the purpose of verifying that the inference algorithm can recover true parameter values. The generating process is explained at the reaction level. For each reaction i in microbiome sample n:

- Metabosystem $Z_{ni} \in \{1,2,\cdots,K\}$ is drawn from the metabosystem mixture θ_n associated with this sample
- Subnetwork $Y_{ni} \in \{1,2,\cdots,L\}$ is drawn from the subnetwork mixture for metabosystem Z_{ni} drawn in the previous step. The mixture coefficients are given in row Z_{ni} of matrix φ (*i.e.*, $\varphi_{Z_{ni}}$)
- For each substrate-product pair in this reaction
  - Choose the substrate from the substrate group for subnetwork Y_{ni}. This compound is sampled from row Y_{ni} of matrix δ (*i.e.*, $\delta_{Y_{ni}}$).
  - Choose the product from the product group for subnetwork Y_{ni}. This compound is sampled from row Y_{ni} of matrix γ (*i.e.*, $\gamma_{Y_{ni}}$).

This generative process is repeated for every reaction in microbiome sample n. The same process generates reactions for all microbiome samples.

To verify that our sampling algorithm can recover the parameter values used for simulation, we have simulated 216 datasets by selecting the number of samples,

compounds, metabosystems and subnetworks from (40, 60, 80, 100), (100, 500, 1000), (3, 5), and (10, 20, 50) respectively. Three different values of 0.05, 0.10 and 0.20 were selected for the concentration parameters (*i.e.*, α_θ, α_φ, α_δ and α_Υ) for the Dirichlet priors in the model. Note that the higher the concentration parameter, the less sparse the corresponding Dirichlet distribution and consequently a more difficult task for the inference algorithm. In our simulations, concentration parameters 0.05 and 0.2 represent the least and the most amount of mixing among metabosystems and subnetworks respectively, as can be seen in Figure 1 below.

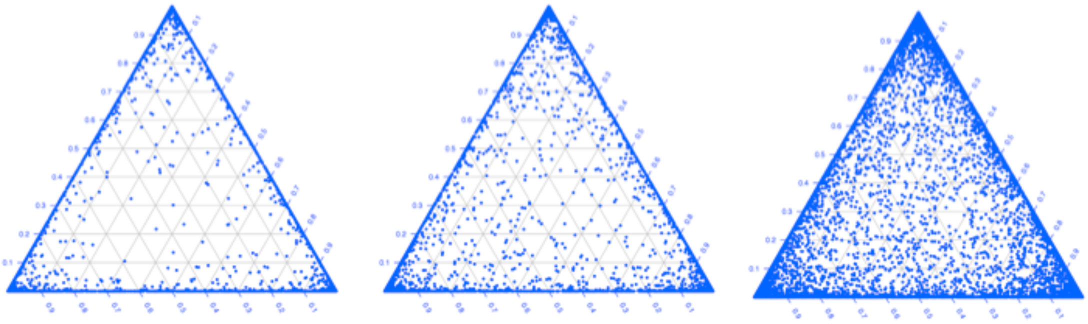

S1: Figure 1 Samples (10,000 independent samples) drawn from Dirichlet distributions with concentration parameters equal to 0.05, 0.1, and 0.2. A significant number of samples drawn from a Dirichlet distribution with concentration parameter of 0.2 show mixed membership to all three groups.

We fitted BiomeNet to the simulated data using the preferred value for the concentration parameters of the Dirichlet priors of the model (0.1), and compared the estimated metabosystem composition with the actual metabosystem composition used to generate the data. We used the Jensen–Shannon divergence (Lin, 1991) to summarize the difference between the estimated distribution of mixing probabilities and the generating

distribution. By using a logarithm in base 2 for the calculations, we obtained values between 0 and 1. On this scale, divergence values of zero indicate exact similarity.

The inference algorithm recovered very good estimates of the mixing probabilities, even when there was a large discrepancy between the specified value concentration parameter (0.01) and the true values for the generating process (2.0). The mean value of the Jensen–Shannon divergence was very close to zero in all cases: 0.0008 when α = 0.05; 0.0018 when α = 0.1, and 0.006 when α = 0.2. The Jensen–Shannon divergence for each of the 216 different scenarios is given in Table 1 below. Given that the estimated metabosystem compositions were very close to the actual metabosystem composition used in a wider variety of scenarios (Table 1 below) there was no need to increase the amount of replication within each scenario. However, we do note that the discrepancies were largest when the gap between the preferred value of concentration parameter (0.01) and the true value (2.0) was very large; putting a distribution on the value of concentration parameter (rather than setting a preferred value) would provide a way of obtaining letting the data influence this value. Nonetheless, our simulations indicate that the inference algorithm is capable of recovering the parameters of the model, and is robust to somewhat large amounts of misspecification.

Lastly, the number of reactions in each network was assumed to follow a Poisson distribution with mean 1000. This choice was intentional. It resulted in networks with far fewer reactions than the two real datasets described in the main text. With more reactions in each network our inference will have more information, making it easier to obtain reliable estimates of model parameters.

Lin J (1991) Divergence measures based on the shannon entropy. *IEEE Transactions on Information Theory* 37(1):145–151.

S1: Table 1. Comparison of estimated and true metabosystem composition for 216 simulated datasets using Jensen-Shannon divergence. Each row represents three simulated networks with the same number of samples, compounds, metabosystems and subnetworks (first four columns) but three different values for the concentration parameters of Dirichlet priors (last three columns). All values are rounded to four decimal places.

Samples	Compounds	Metabosystems	Subnetworks	Concentration Parameter		
				0.05	0.10	0.20
				Jensen-Shannon Divergence		
40	100	3	10	0.0001	0.0003	0.0091
40	100	3	20	0.0012	0.0005	0.0039
40	100	3	50	0.0003	0.0006	0.0020
40	100	5	10	0.0006	0.0022	0.0187
40	100	5	20	0.0004	0.0016	0.0093
40	100	5	50	0.0008	0.0018	0.0065
40	500	3	10	0.0004	0.0039	0.0025
40	500	3	20	0.0006	0.0013	0.0023
40	500	3	50	0.0004	0.0006	0.0022
40	500	5	10	0.0026	0.0048	0.0135
40	500	5	20	0.0015	0.0022	0.0100
40	500	5	50	0.0003	0.0017	0.0066
40	1000	3	10	0.0005	0.0011	0.0011
40	1000	3	20	0.0003	0.0011	0.0025
40	1000	3	50	0.0002	0.0006	0.0019
40	1000	5	10	0.0036	0.0042	0.0075
40	1000	5	20	0.0009	0.0022	0.0141
40	1000	5	50	0.0006	0.0017	0.0095
60	100	3	10	0.0002	0.0004	0.0039
60	100	3	20	0.0008	0.0004	0.0030
60	100	3	50	0.0004	0.0006	0.0019
60	100	5	10	0.0006	0.0024	0.0199
60	100	5	20	0.0004	0.0016	0.0089
60	100	5	50	0.0010	0.0016	0.0099
60	500	3	10	0.0003	0.0048	0.0026
60	500	3	20	0.0007	0.0016	0.0027
60	500	3	50	0.0002	0.0005	0.0021
60	500	5	10	0.0008	0.0056	0.0129
60	500	5	20	0.0029	0.0017	0.0064
60	500	5	50	0.0005	0.0014	0.0058
60	1000	3	10	0.0003	0.0017	0.0016
60	1000	3	20	0.0002	0.0010	0.0030
60	1000	3	50	0.0002	0.0006	0.0017
60	1000	5	10	0.0027	0.0036	0.0069
60	1000	5	20	0.0005	0.0051	0.0129
60	1000	5	50	0.0005	0.0016	0.0065
80	100	3	10	0.0002	0.0006	0.0035

80	100	3	20	0.0009	0.0004	0.0031
80	100	3	50	0.0004	0.0006	0.0023
80	100	5	10	0.0006	0.0036	0.0227
80	100	5	20	0.0004	0.0017	0.0091
80	100	5	50	0.0009	0.0014	0.0080
80	500	3	10	0.0003	0.0039	0.0017
80	500	3	20	0.0007	0.0009	0.0029
80	500	3	50	0.0003	0.0006	0.0022
80	500	5	10	0.0063	0.0052	0.0114
80	500	5	20	0.0021	0.0022	0.0059
80	500	5	50	0.0007	0.0016	0.0084
80	1000	3	10	0.0008	0.0017	0.0016
80	1000	3	20	0.0002	0.0010	0.0030
80	1000	3	50	0.0002	0.0006	0.0015
80	1000	5	10	0.0019	0.0043	0.0076
80	1000	5	20	0.0007	0.0019	0.0135
80	1000	5	50	0.0004	0.0015	0.0050
100	100	3	10	0.0002	0.0006	0.0040
100	100	3	20	0.0006	0.0004	0.0032
100	100	3	50	0.0004	0.0005	0.0022
100	100	5	10	0.0007	0.0030	0.0173
100	100	5	20	0.0004	0.0019	0.0085
100	100	5	50	0.0009	0.0015	0.0087
100	500	3	10	0.0003	0.0031	0.0019
100	500	3	20	0.0007	0.0013	0.0028
100	500	3	50	0.0003	0.0006	0.0023
100	500	5	10	0.0005	0.0039	0.0103
100	500	5	20	0.0026	0.0022	0.0064
100	500	5	50	0.0005	0.0016	0.0056
100	1000	3	10	0.0004	0.0012	0.0038
100	1000	3	20	0.0002	0.0007	0.0031
100	1000	3	50	0.0001	0.0005	0.0015
100	1000	5	10	0.0026	0.0033	0.0074
100	1000	5	20	0.0005	0.0017	0.0122
100	1000	5	50	0.0004	0.0017	0.0052
