# Supplementary material for: BiomeNet: A Bayesian Model for Inference of Metabolic Divergence among Microbial Communities
Source: PLoS Comput Biol. 2014 Nov 20;10(11):e1003918. doi: 10.1371/journal.pcbi.1003918 (PMC4238953; doi:10.1371/journal.pcbi.1003918)
Supplement: Text S2 — Analytical solutions for the individual terms of the posterior distribution of the latent variables. For inference under BiomeNet we sample from the posterior distribution of latent variables given the data. We use collapsed Gibbs sampling by integrating out the latent variables θ, φ, δ, γ and sample from the posterior distributions of the metabosystem (Z) and subnetwork (Y) assignments for each reaction conditional on the assignments of all other reactions. This is a high dimensional distribution, and the analytical solutions for the individual terms are given in these notes. (PDF) [file pcbi.1003918.s002.pdf]

Text S2: Analytical solutions for the individual terms of the posterior distribution of the latent variables.

We assume that each microbiome sample is a mixture of K metabosystems, with different mixing probabilities for different samples. The relative contribution of each metabosystem to the microbiome associated with the nth sample is modeled through latent variable θ_n, a probability vector of K values summing to one.

Each metabosystem is comprised of a fixed number (L) of metabolic subnetworks. The contribution of each subnetwork to the kth metabosystem, φ_k, is modeled by a vector of L mixing probabilities that sum to one. The element φ_{kl} in a $K \times L$ matrix φ represents the relative contribution of subnetwork l in metabosystem k.

Each subnetwork is comprised of a mixture of reactions, which are linked through shared chemical compounds. Thus, each subnetwork has its own substrate (S) group and product (R) group. Note that compound participating in intermediary reactions can belong to both the substrate and the product group. For subnetwork l, we denote the substrate group as δ_l and the product group as γ_l, each a vector of C probability values summing to one, where C is the number of compounds. With L subnetworks, there are two $L \times C$ matrices δ and γ, one for substrates and one for products respectively. The value in row l and column c of matrix δ represents the relative contribution of compound c in the substrate group of subnetwork l. A similar definition applies for product groups in matrix γ.

We assume an independent and identical (iid) sparse symmetric Dirichlet prior on θ_n, and the rows of φ, δ and γ:

$$
\begin{aligned}
\theta_n &\sim Dirichlet(\alpha_\theta) \\
\varphi_k &\sim Dirichlet(\alpha_\varphi) \\
\delta_l &\sim Dirichlet(\alpha_\delta) \\
\gamma_l &\sim Dirichlet(\alpha_\gamma)
\end{aligned}
$$

The complete likelihood of the data given the hyper-parameters of the prior distributions is:

$$P\left(Z,Y,R,S,\theta,\varphi,\delta,\gamma \mid \alpha_\theta,\alpha_\varphi,\alpha_\delta,\alpha_\gamma\right)=$$
$$\prod_{n=1}^{N}\prod_{i=1}^{I_n} P\left(Z_{ni} \mid \theta_n\right) P\left(Y_{ni} \mid \theta_{Z_{ni}}\right)$$
$$\prod_{n=1}^{N}\prod_{i=1}^{I_n}\prod_{j=1}^{J_{ni}} P\left(S_{nij} \mid \delta_{Y_{ni}}\right) P\left(R_{nij} \mid \gamma_{Y_{ni}}\right)$$
$$\prod_{n=1}^{N} P\left(\theta_n \mid \alpha_\theta\right)\prod_{k=1}^{K} P\left(\varphi_k \mid \alpha_\varphi\right)\prod_{l=1}^{L}\left[P\left(\delta_l \mid \alpha_\delta\right)P\left(\gamma_l \mid \alpha_\delta\right)\right]$$

where N is the total microbiome samples, I_n is the total number of reactions in the metabolic network associated the nth microbiome sample and J_{ni} is the total number of substrate-product pairs in in the ith reaction of the nth sample. Z and Y represent the metabosystem and subnetwork assignments for all reactions in all samples, and as Z_{ni} and Y_{ni} represent the metabosystem and subnetwork assignments of reaction i within sample n.

To infer the model framework, we need to sample from the posterior distribution of latent variables given the data:

$$P\left(Z,Y,\theta,\varphi,\delta,\gamma \mid R,S,\alpha_\theta,\alpha_\varphi,\alpha_\delta,\alpha_\gamma\right)$$

where Z, Y, θ, φ, δ, and γ are latent variables in the model. We use collapsed Gibbs sampling to integrate out the other latent variables θ, φ, δ, γ and sample from the posterior distributions of the metabosystem (Z) and subnetwork (Y) assignments for each reaction conditional on the assignments of all other reactions. We use the following conditional probability to sample the subnetwork and metabosystem assignment of one reaction in a microbiome sample given we know the subnetwork and metabosystem assignments of all other reactions in every microbiome sample:

$$P\left(Z_{ni},Y_{ni} \mid S,R,Z_{-ni},Y_{-ni}\right)=$$

$$\frac{\iiiint P\left(S,R,Z,Y,\theta,\varphi,\delta,\gamma\right)d\theta\, d\varphi\, d\delta\, d\gamma}{\iiiint P\left(S,R,Z_{-ni},Y_{-ni},\theta,\varphi,\delta,\gamma\right)d\theta\, d\varphi\, d\delta\, d\gamma}=$$

$$\frac{\int P(Z \mid \theta)P\left(\theta \mid \alpha_\theta\right)d\theta}{\int P\left(Z_{-ni} \mid \theta\right)P\left(\theta \mid \alpha_\theta\right)d\theta}\times\frac{\int P\left(Y \mid Z,\varphi\right)P\left(\varphi \mid \alpha_\varphi\right)d\varphi}{\int P\left(Y_{-ni} \mid Z_{-ni},\varphi\right)P\left(\varphi \mid \alpha_\varphi\right)d\varphi}$$

$$\times\frac{\int P\left(S \mid Y,\delta\right)P\left(\delta \mid \alpha_\delta\right)d\delta}{\int P\left(S \mid Y_{-ni},\delta\right)P\left(\delta \mid \alpha_\delta\right)d\delta}\times\frac{\int P\left(R \mid Y,\gamma\right)P\left(\gamma \mid \alpha_\gamma\right)d\gamma}{\int P\left(R \mid Y_{-ni},\gamma\right)P\left(\gamma \mid \alpha_\gamma\right)d\gamma}$$

where Z_{-ni} and Y_{-ni} denote the metabosystem and subnetwork assignment for all reactions in all samples except only reaction *i* in sample *n*. The analytical solutions for the individual terms of the above equation are:

$$\int P(Z \mid \theta)P\left(\theta \mid \alpha_\theta\right)d\theta=$$

$$=\int\prod_{n=1}^{N}\prod_{i=1}^{I_n}P\left(Z_{ni} \mid \theta_n\right)\prod_{n=1}^{N}P\left(\theta_n \mid \alpha_\theta\right)d\theta$$

$$=\int\prod_{n=1}^{N}\prod_{k=1}^{K}\theta_{nk}^{G_n^k}\prod_{n=1}^{N}\frac{\Gamma\left(K\alpha_\theta\right)}{\Gamma\left(\alpha_\theta\right)^K}\prod_{k=1}^{K}\theta_{nk}^{\alpha_{\theta-1}}d\theta$$

$$=\prod_{n=1}^{N}\frac{\Gamma\left(K\alpha_\theta\right)}{\Gamma\left(\alpha_\theta\right)}\frac{\prod_{k=1}^{K}\Gamma\left(\alpha_\theta+G_n^k\right)}{\Gamma\left(\sum_{k=1}^{K}\left(\alpha_\theta+G_n^k\right)\right)}$$

where G_n^k is the number of times the nth sample is assigned to the kth metabosystem.

$$\int P(Y \mid Z,\varphi)P(\varphi \mid \alpha_{\varphi})d\varphi =$$

$$= \int \prod_{n=1}^{N}\prod_{i=1}^{I_n} P(Y_{ni} \mid Z_{ni},\varphi)\prod_{k=1}^{K} P(\varphi_k \mid \alpha_{\varphi})d\varphi$$

$$= \int \prod_{k=1}^{K}\prod_{l=1}^{L} \varphi_{kl}^{G_k^l} \prod_{k=1}^{K} \frac{\Gamma(L\alpha_{\varphi})}{\Gamma(\alpha_{\varphi})^L} \prod_{l=1}^{L} \varphi_{kl}^{\alpha_{\varphi}-1} d\varphi$$

$$= \prod_{k=1}^{K} \frac{\Gamma(L\alpha_{\varphi})}{\Gamma(\alpha_{\varphi})} \frac{\prod_{l=1}^{L}(\alpha_{\varphi}+G_k^l)}{\Gamma\left(\sum_{l=1}^{L}(\alpha_{\varphi}+G_k^l)\right)}$$

where G_k^l is the number of times a reaction is assigned to the lth subnetwork for a sample in the kth metabosystem.

$$\int P(S \mid \delta,Y)P(\delta \mid \alpha_{\delta})d\delta =$$

$$= \int \prod_{n=1}^{N}\prod_{i=1}^{I_n}\prod_{j=1}^{J_{ni}} P(S_{nij} \mid \delta_{Yni})\prod_{l=1}^{L} P(\delta_l \mid \alpha_{\delta})d\delta$$

$$= \int \prod_{l=1}^{L}\prod_{c=1}^{C} \delta_{lc}^{SG_c^l} \prod_{l=1}^{L} \frac{\Gamma(C\alpha_{\delta})}{\Gamma(\alpha_{\delta})^C} \prod_{C=1}^{C} \delta_{lc}^{\alpha_{\delta}-1} d\delta$$

$$= \prod_{l=1}^{L} \frac{\Gamma(C\alpha_{\delta})}{\Gamma(\alpha_{\delta})^C} \frac{\prod_{c=1}^{C}\Gamma(\alpha_{\delta}+SG_c^l)}{\Gamma\left(\sum_{c=1}^{C}(\alpha_{\delta}+SG_c^l)\right)}$$

where SG_c^l is the number of times the cth compound is assigned to the *substrate* group of the lth subnetwork.

$$\int P(R \mid \delta, Y) P\left(\gamma \mid \alpha_\gamma\right) d\gamma =$$

$$= \int \prod_{n=1}^{N} \prod_{i=1}^{I_n} \prod_{j=1}^{J_{ni}} P\left(R_{nij} \mid \gamma_{Yni}\right) \prod_{l=1}^{L} P\left(\gamma_l \mid \alpha_\gamma\right) d\gamma$$

$$= \int \prod_{l=1}^{L} \prod_{c=1}^{C} \delta_{lc}^{RG_c^l} \prod_{l=1}^{L} \frac{\Gamma\left(C\alpha_\gamma\right)}{\Gamma\left(\alpha_\gamma\right)^C} \prod_{C=1}^{C} \delta_{lc}^{\alpha_\gamma - 1} d\gamma$$

$$= \prod_{l=1}^{L} \frac{\Gamma\left(C\alpha_\gamma\right)}{\Gamma\left(\alpha_\gamma\right)^C} \frac{\prod_{c=1}^{C} \Gamma\left(\alpha_\gamma + RG_c^l\right)}{\Gamma\left(\sum_{c=1}^{C}\left(\alpha_\gamma + RG_c^l\right)\right)}$$

where RG_c^l is the number of times the cth compound is assigned to the *product* group of the lth subnetwork
