# Supplementary material for: BiomeNet: A Bayesian Model for Inference of Metabolic Divergence among Microbial Communities
Source: PLoS Comput Biol. 2014 Nov 20;10(11):e1003918. doi: 10.1371/journal.pcbi.1003918 (PMC4238953; doi:10.1371/journal.pcbi.1003918)
Supplement: Text S3 — Characterizing the reaction composition of a metabosystem, and measuring compositional differences between metabosystems. A detailed description of the method for characterizing the composition of a metabosystem at the level of its metabolic reactions, the method for measuring compositional differences between metabosystems, and the method for assessing robustness of metabosystems to the L value. The criteria for classifying principal subnetworks and discriminatory subnetworks are also provided. (PDF) [file pcbi.1003918.s003.pdf]

Text S3: Characterizing the reaction composition of a metabosystem, and measuring compositional differences between metabosystems.

Characterizing the composition of a metabosystem at the level of its metabolic reactions

To investigate the impact of the chosen value of L on the metabosystems, and to ensure that a large enough value has been chosen, the composition of each metabosystem must be characterized at the reaction level. To characterize the reaction composition of a metabosystem we must combine information about the reaction composition of the subnetworks that contribute to that metabosystem.

The information about each subnetwork's reaction composition is contained in an R by L reaction matrix of mixing probabilities, where R is the number of unique reactions in the data and L is the chosen number of subnetworks in the model. For each column in the matrix (*i.e.*, subnetwork), we take each reaction's mixing probability and weight it according to that subnetwork's mixing probability for a given metabosystem. This yields a weighted vector of size R for each subnetwork.

To combine the reaction contributions of all subnetworks, we sum the weighted contribution of each reaction to a given metabosystem. This yields a single vector of size R for that metabosystem, which is then normalized. Such a vector is computed for each of the K metabosystems in the model.

Measuring compositional differences between metabosystems

Characterizing the reaction composition of each metabosystem as a single vector of mixing probabilities allows us to quantify their divergence in terms of the uniqueness of their characteristic reaction profiles. Specifically, we employed the Jenson-Shannon Divergence (JSD) measure, which provides a symmetric measure of the difference between two probability distributions (Lin, 1991).

For a fixed value of K=3, we applied BiomeNet to the Muegge et al. (2011) dataset using five L values = 50, 100, 150, 200 and 250. This dataset had R = 2,824 unique reactions. We characterized the composition of the R reactions within each of the three metabosystems for the five different values for L. We computed JSD scores for all pairwise comparisons between metabosystems, and also between

different values of *L*. This yielded a divergence matrix for each of the 6 ways to compare metabosystems, each one being a 5×5 matrix of JSD scores. Results are shown in Figure 4 of the main text.

Also using *K*=3, we applied BiomeNet to the Qin et al. (2010) dataset using four *L* values = 50, 100, 150 and 200. This dataset had *R* = 3,433 unique reactions. We characterized the composition of the *R* reactions within the three metabosystems for the four different values for *L*. As above, we computed JSD scores for all pairwise comparisons between metabosystems and between different values of *L*. This yielded a divergence matrix for each of the 6 ways to compare metabosystems, each one being a 4×4 matrix of JSD scores. Results are shown in Figure 6 of the main text.

Assessing robustness of metabosystems to the L value, and avoiding unnecessarily large values for of K

Comparing the reaction profiles of metabosystems via JSD scores provide information that is relevant to (*i*) verifying that the composition of reactions within the *K* different metabosystems is robust to *L*, and (*ii*) identifying the minimum value of *L*.

If *L* is large enough, and the model has resolved discriminatory subnetworks, then the reaction composition of those subnetworks should lead to characteristic profiles for the metabosystems. If the inferred reaction profiles are robust to the value of *L*, then metabosystems having the same characteristic reactions should have very low JSD scores. This can be most easily visualized as a heat map of pairwise JSD comparisons. JSD comparisons within matrices along the diagonal in Figures 4 and 6 in the main text represent such comparisons.

Avoiding unnecessarily large values of *K* reduces computational costs and aids the interpretability of results. An analytical objective of BiomeNet is to infer a characteristic reaction profile for each of the *K* metabosystems. When *K* is larger than necessary, the reaction composition of some metabosystems will be very similar and metabosystems will fall into fewer than *K* groups; *i.e.*, redundant metabosystems will have relatively small differences in reaction composition, and

those differences will be of similar magnitude to differences observed for comparisons involving the same metabosystem. This can be visualized by comparing pairwise JSD scores for comparisons within clusters of metabosystems to comparisons of JSD scores between the clusters of metabosystems.

For both real datasets we analyzed with BiomeNet we selected the value of K according to biological criteria ($K = 3$ in both cases). In both cases, divergence in reaction profiles was sufficiently stable to easily coordinate metabosystems across analyses under different values of L (Text S6 and figures 1 and 2 in Text S6). Having easily resolved the structure of the metabosystems, we constructed separate matrices of JSD scores for comparisons within and between the three metabosystems. This yielded 3 within-metabosystem divergence matrices and 3 between-metabosystem divergence matrices. These are organized into two large heat maps (one for each dataset) presented in Figures 4 and 6 in the main text. Within these heat maps divergence matrices along the diagonal represent comparisons within the same metabosystems, and the off-diagonal matrices are for comparisons between metabosystems.

Inferring principal subnetworks and discriminatory subnetworks under BiomeNet

As long as the number of subnetworks in the model, L, is not too small we expect that inferences under BiomeNet should be relatively robust to its chosen value. This is because there will be "*redundant subnetworks*" when L is larger than needed; *i.e.*, they will carry very little weight (posterior mixing probability). Thus, the reactions that comprise a redundant subnetwork will have only a trivial impact on the reaction composition of each metabosystem. Because we expect redundant subnetworks, a capacity to identify them (and filter them out) will facilitate the interpretation of results when the L value is large. We adopted a cut-off value for redundant subnetworks to be a posterior mixing probability less than 2× the probability of selecting a subnetwork at random ($1/L$). Subnetworks having larger mixing probabilities than this cutoff are referred to as the "*principal subnetworks*" of the metabosystem.

For some datasets there could be many principal subnetworks, and some of these can have high, but very similar, mixing probabilities in all the metabosystems; thus, some of the principal subnetworks do not contribute much to the differences between metabosystems (*i.e.*, these are shared subnetworks). Such subnetworks are hereafter referred to as "*non-discriminatory subnetworks*". Again, to facilitate the interpretation of results we adopted the following criterion for identifying highly "*discriminatory subnetworks*": a ratio of its membership probability to a selected metabosystems to the largest probability in any other metabosystem >10. Formally:

$$\left| \log\left(\frac{p(k_1)}{\max\left\{ p(k_2)\ldots p(k_n) \right\}} \right) \right| > 1$$

Only the principal subnetworks were scored as discriminatory according to the above criterion.

References:

1. Qin J, et al. (2010) A human gut microbial gene catalogue established by metagenomic sequencing. Nature 464:59-65.
2. Muegge B, et al. (2011) Diet drives convergence in gut microbiome functions across mammalian phylogeny and within humans. Science 332:970-973.
3. Lin J (1991) Divergence measures based on the Shannon Entropy. IEEE Transaction on Information Theory 37:145-151.
