# Supplementary material for: BiomeNet: A Bayesian Model for Inference of Metabolic Divergence among Microbial Communities
Source: PLoS Comput Biol. 2014 Nov 20;10(11):e1003918. doi: 10.1371/journal.pcbi.1003918 (PMC4238953; doi:10.1371/journal.pcbi.1003918)
Supplement: Text S4 — PCoA analysis of mammalian microbiome samples. PCoA analysis of Enzyme Commission assignments and KEGG Orthology assignments for the mammalian dataset reveals a separation between carnivore and herbivore gut microbiomes. (PDF) [file pcbi.1003918.s004.pdf]

Text S4: PCoA analysis of mammalian microbiome samples.

We also carried out PCoA analysis of the Enzyme Commission (EC) and KEGG orthology assignments for the mammalian dataset (Muegge, et al., 2011). The data were obtained from MGRAST, and PCoA was applied to Bray-Curtis dissimilarity scores (Bray and Curtis, 1957). The results are presented in Figures 1 and 2 below.

Similar to BiomeNet, PCoA is identifies a clear separation between the carnivore (red) and herbivore (green) samples. Also similar to BiomeNet, PCoA suggest that some omnivore micorbiomes may be more similar to carnivores and others more similar to herbivores. However, PCoA and other similar methods do not lend themselves to interpretation and therefore the nature of this separation visualized in Figures 1 and 2 below cannot be easily explained using that method. There have been efforts to interpret principal components or dimensions discovered by methods in the PCA family (Gu and Chipman, 2005) but they often require a post-hoc processing of the results produced by those methods. By providing a framework for explaining and interpreting the discovered patterns (presented in main text), BiomeNet provides a valuable supplement to the PCA family of methods.

Bray, J. R. and J. T. Curtis. 1957. An ordination of upland forest communities of southern Wisconsin. Ecological Monographs 27:325-349.

Gu H, Chipman H (2005) Interpretable dimension reduction. *Journal of Applied Statistics* 32(9):969-987.

Muegge B, *et al.* (2011) Diet drives convergence in gut microbiome functions across mammalian phylogeny and within humans. *Science* 332:970-973.

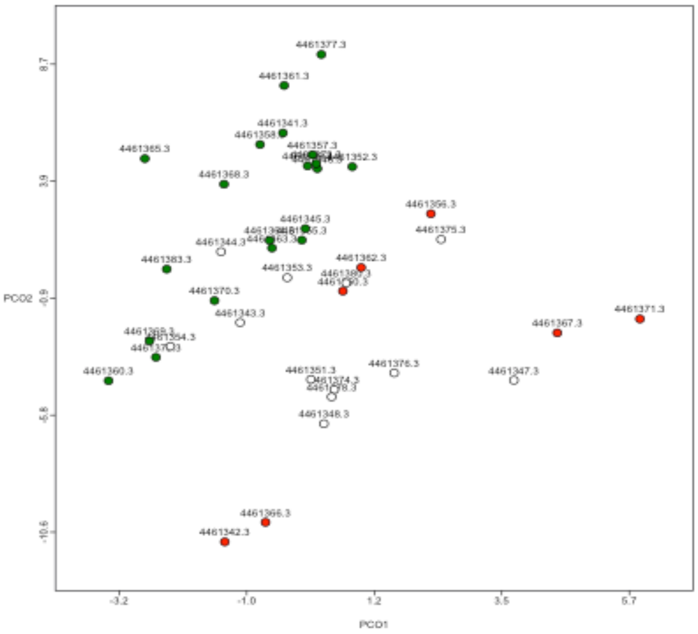

S4: Figure 1 PCoA analysis of EC subsystem data for samples from 33 species of mammals. Red dots represent carnivores, green dots herbivores and open circles omnivores.

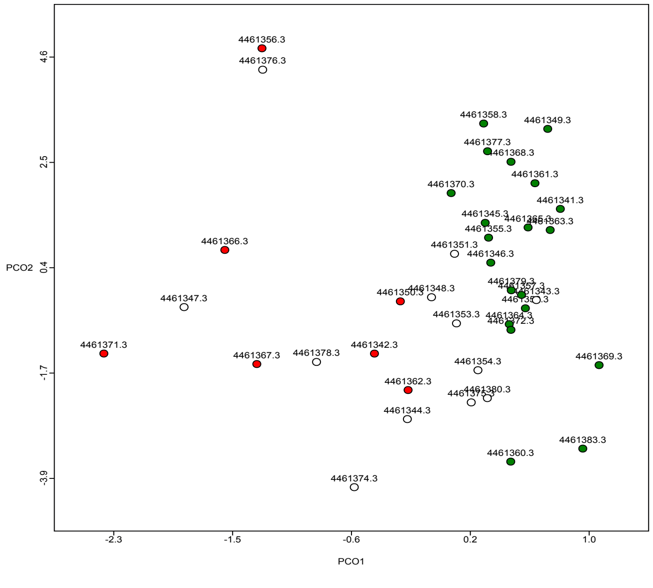

S4: Figure 2 PCoA analysis of KO data from 33 species of mammals (4). Red dots represent carnivores, green dots herbivores and open circles omnivores.
