# Supplementary material for: BiomeNet: A Bayesian Model for Inference of Metabolic Divergence among Microbial Communities
Source: PLoS Comput Biol. 2014 Nov 20;10(11):e1003918. doi: 10.1371/journal.pcbi.1003918 (PMC4238953; doi:10.1371/journal.pcbi.1003918)
Supplement: Text S5 — Source and processing of metagenomic data. The source of the two gut metagenome datasets and a description how those data were processed to obtain abundance values for substrate-product pairs that were input into the model. (PDF) [file pcbi.1003918.s005.pdf]

**Text S5: Source and processing of metagenomic data.**

Enzyme abundances for the 38 mammalian metagenomes (Muegge, *et al.* 2011) were obtained from MG-RAST (Meyer *et al*. 2008) (project #116, using SEED subsystem). We used the KEGG database to map Enzyme Commission (EC) numbers to reactions. Where an EC number mapped to multiple reactions, we applied abundance counts to each reaction. We decompose each reaction into its substrate-product pairs (Fig. 1), using only those pairs denoted as RPAIRS in KEGG database. The abundance calculated for each reaction is viewed as the latent capacity of a metagenome for that reaction.

To allow the model to take full advantage of the connectivity and dependence structure in the network, we utilized all categories of RPAIRS. A consequence is that unnecessary connectivity information is included in the data; an example is a currency compound such as water. As our model uses co-occurrence information extracted from the abundance data to infer subnetworks, thus unnecessary RPAIRs are not expected to bias the subnetwork formation. Although the currency compounds do represent a source of noise, they represent a small fraction of RPAIRS, and restricting the data to, say, just the "main" RPAIRS category deprives the model from a substantial source of connectivity information.

Enzyme abundance data was not available for the 124 adult human gut microbiome samples of Qin *et al*. (2010), therefore, we computed the abundance from the counts of reads that could be assigned an EC number. The assembled reads and predicted genes were obtained from http://gutmeta.genomics.org.cn. We used SOAPaligner v 2.20 to map the reads back to contigs by using the parameter values

reported by Qin *et al*. (2010). We estimated gene-level abundance according to the method in Arumugam *et al.* (2011). If $R$ is the set of all assembled reads $r$ that have an overlap with the locus of predicted protein $g$ in a contig, then the abundance of $g$ is calculated as follows:

$$abundance(g) = \sum_{r \in R} \frac{base\ overlap(g,r)}{base\ length(g)}$$

To assign EC numbers to genes, we used the assignments of proteins to KEGG orthologous groups (KO) made by Qin et al. (2010), using the KEGG database to map KO groups to EC numbers. Sometimes a KO group would map to more than one EC number. EC abundance was computed as a sum over the abundances of all members of KO groups that mapped to an EC. Lastly, we decompose the enzyme-catalyzed reactions as described above for the mammalian dataset. Two samples sequenced at a very different level and via a different method (MH0006 and MH0012) were excluded from the analysis.

BiomeNet takes as input a plain text file with each reaction decomposed into counts for substrate-product pairs. The program does not apply normalization to those data; the user must do this if it is warranted. KEGG compound numbers are used to denote a specific substrate-product pair, and a KEGG reaction number is used to denote the reaction to which a given pair of compounds belongs. The fully processed data, formatted for input into BiomeNet is available along with the source code at (http://sourceforge.net/projects/biomenet/).

Arumugam M, *et al.* (2011) Enterotypes of the human gut microbiome. *Nature* 473:174-180.

Meyer, F; Paarmann, D; D'Souza, M; Olson, R; Glass, EM; Kubal, M; Paczian, T; Rodriguez, A; Stevens, R; Wilke, A; Wilkening, J; Edwards, RA (2008). "The metagenomics RAST server – a public resource for the automatic phylogenetic and functional analysis of metagenomes". *BMC Bioinformatics* **9** (1): 386.

Muegge B, *et al.* (2011) Diet drives convergence in gut microbiome functions across mammalian phylogeny and within humans. *Science* 332:970-973.

Qin J, *et al.* (2010) A human gut microbial gene catalogue established by metagenomic sequencing. *Nature* 464:59-65.
