# Supplementary material for: BiomeNet: A Bayesian Model for Inference of Metabolic Divergence among Microbial Communities
Source: PLoS Comput Biol. 2014 Nov 20;10(11):e1003918. doi: 10.1371/journal.pcbi.1003918 (PMC4238953; doi:10.1371/journal.pcbi.1003918)
Supplement: Text S6 — Robustness of metabosystem composition to number of subnetworks. Evaluation of alternative numbers of subnetworks (L) in the model reveals that the reaction composition of the metabosystems is relatively stable to the specified value of L. (PDF) [file pcbi.1003918.s006.pdf]

Text S6: Robustness of metabosystem composition of samples to number of subnetworks, and coordination of metabosystems across different runs.

As mentioned in the main text, BiomeNet assigns arbitrary numbers as metabosystem labels. Because BiomeNet is unsupervised, different runs can potentially yield different numbers to label metabosystems having common biological underpinnings. This is common with any clustering algorithm or mixture model, and care must be taken when attempting to coordinate metabosystems obtained from different runs of BiomeNet. However, when the metabosystems inferred by BiomeNet have characteristic reaction profiles that are stable across analyses (see Text S3 for measurement of the reaction composition of a metabosystem), then the mixing probabilities of the metabosystems for each sample will also be stable. This means that their relative positions in a plot (*e.g.*, the simplex plots in the main text) will be stable, and the labels for metabosystems can be visually coordinated if K is not too large.

Results presented in the main text were based on setting the number of subnetworks (L) to 100 for both datasets. However, we also analyzed under a model with different values for L (50, 150 and 200), and comparison of results across these analyses requires that we coordinate their reaction system labels. Since we used K=3, we can coordinate the labels visually by rotating the simplex plots until the distributions of samples within the simplex match up. This approach will only work for across different values of L if its value is large enough and the characteristic reaction composition of the metabosystems are stable. This was indeed the case for both real datasets.

Figure 1 below shows that the metabosystem composition of the 38 samples of the mammal dataset is relatively stable with regards to the choice of L; that is, the relative

distribution of the samples within the simplex is remarkably similar after appropriate rotation of the plots. Good segregations of carnivore samples (magenta dots) and herbivore samples (green dots) was largely unaffected by the value of L, although when $L \geq 100$ is characterized by remarkable consistency of the distribution of samples within the simplex. Of course, the model is free to determine the subnetwork composition of the metabosystems, and the contribution of reactions to those subnetworks could differ; however, as we show elsewhere (main text and Text S3) the constituent reactions of these metabosystems also is robust as long the value of L was ≥ 100.

Figure 2 below shows that the metabosystem composition of the 124 samples of adult human gut samples is relatively stable within the simplex as long as L was ≥ 100. These data differ form the mammal data in that the sample labels were not well separated within the simplex when $L = 50$. However, for $L \geq 100$, we do observe one axis of separation; the contribution of metabosystem 2 tends to be higher in IBD samples (red) and lower in healthy samples (green). Further, the relative positions of each sample was remarkably similar for $L \geq 100$, as well as the constituent reactions of the metabosystems (main text and Text S3).

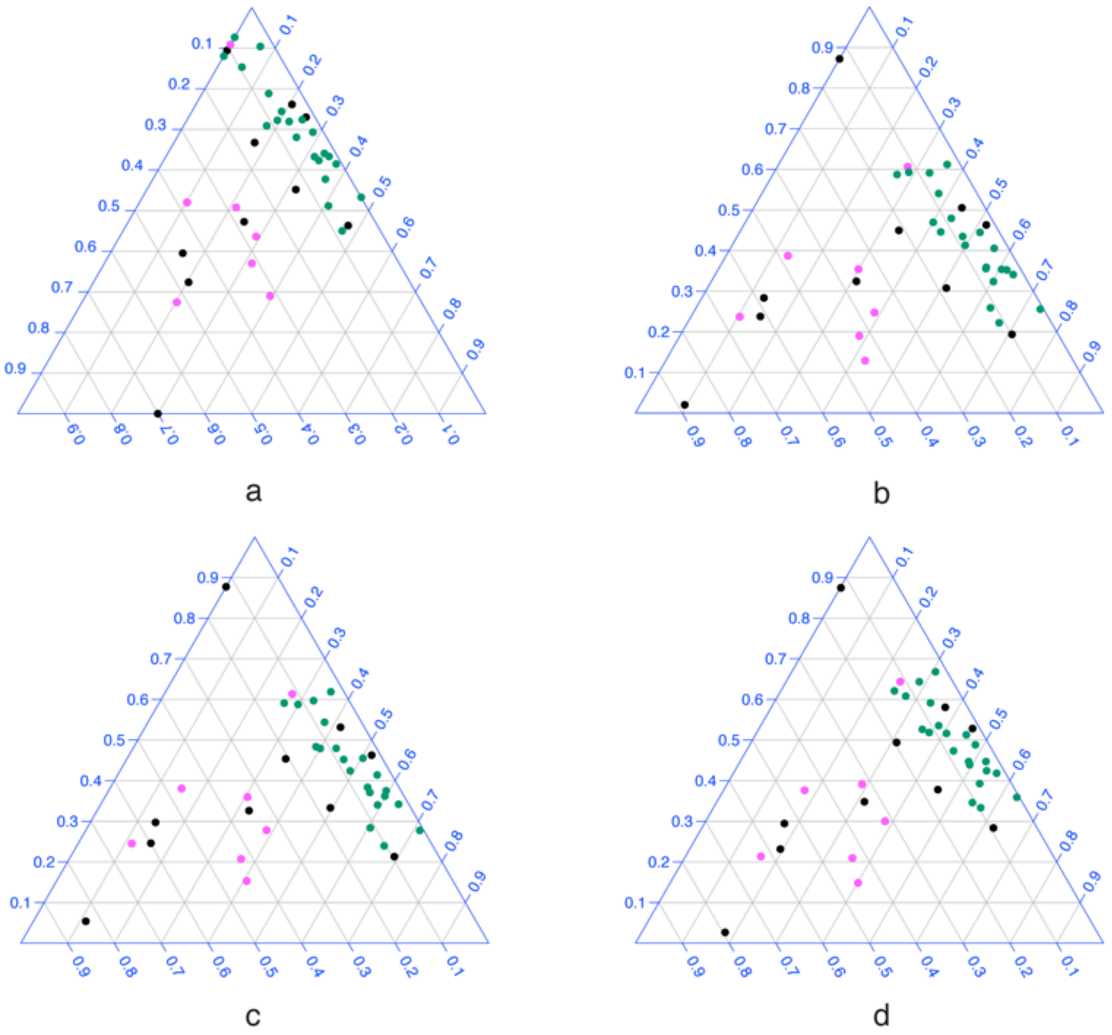

S6: Figure 1 Metabosystem predictions for the mammal dataset samples when the model is trained with varying number of subnetworks (L). (a) L=50, (b) L=100 (equivalent to analysis in main paper), (c) L=150 and (d) L=200. Because the model arbitrarily sets numerical labels assigned to the metabosystems, the labels for metabosystems were switched For L=50 so as to coordinate the results across the analyses. Note that plot (a) has been rotated 120 degrees counter-clockwise to reveal the same composition of metabosystems for this case compared to the other 3 cases. Each sample is colored according to it's a prior label for ecological niche: magenta for carnivore, green for herbivore, and black for omnivore.

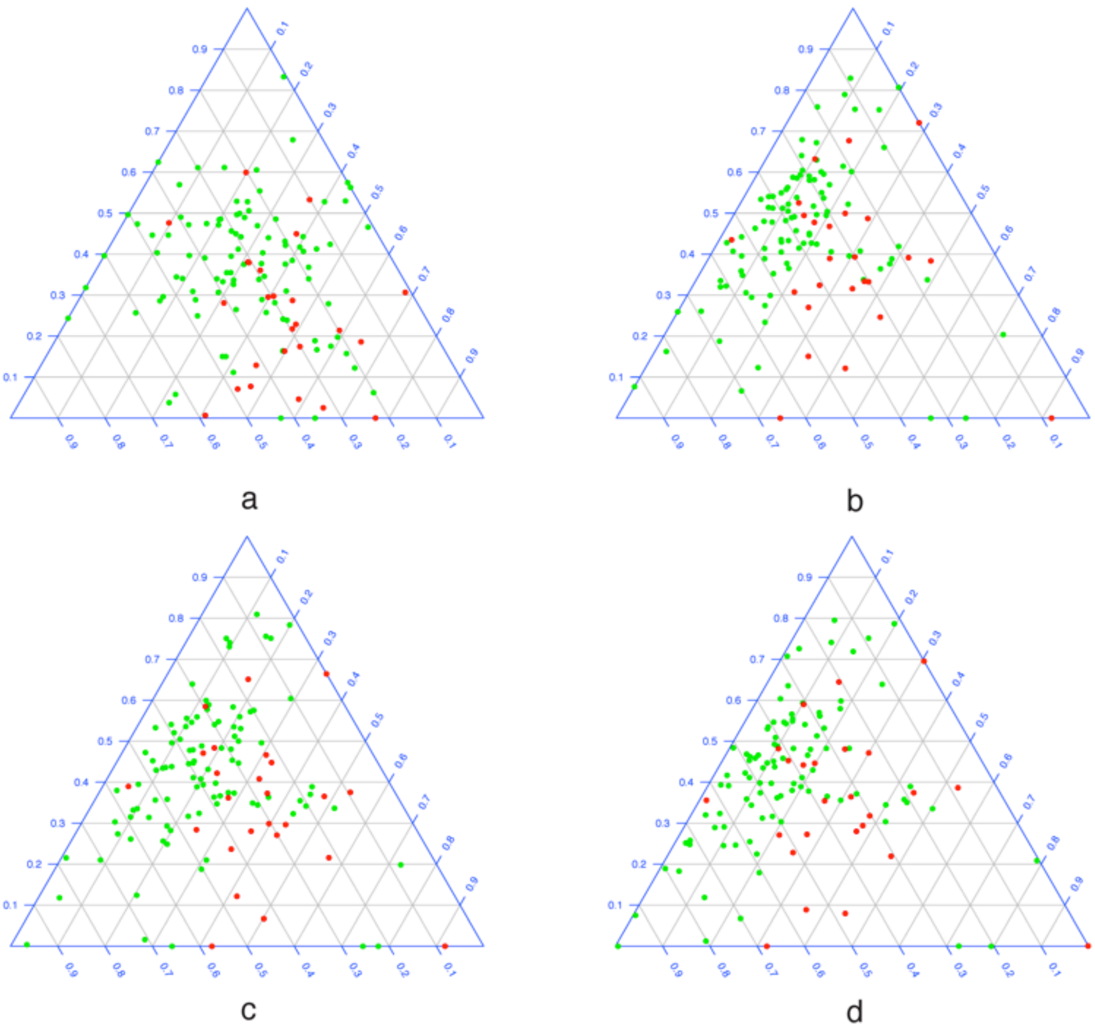

S6: Figure 2 Metabosystem predictions for the IBD/healthy dataset samples when the model is trained with varying number of subnetworks (L). (a) L=50, (b) L=100 (equivalent to data in main paper), (c) L=150 and (d) L=200. Each sample is colored according to it's a prior label for health status: red is for IBD patient and green is for a healthy adult.
