# Supplementary material for: BiomeNet: A Bayesian Model for Inference of Metabolic Divergence among Microbial Communities
Source: PLoS Comput Biol. 2014 Nov 20;10(11):e1003918. doi: 10.1371/journal.pcbi.1003918 (PMC4238953; doi:10.1371/journal.pcbi.1003918)
Supplement: Text S7 — Subnetwork composition of mammalian metabosystems. A presentation of the inferred contribution of 100 subnetworks to three metabosystems in the model, and the identification of the most discriminatory subnetwork for metabosystem 1. (PDF) [file pcbi.1003918.s007.pdf]

Text S7: Contribution of all subnetworks to mammalian metabosystems.

Figure 1 below presents the composition ribbon for all 100 subnetworks for the mammalian gut microbiome samples. Only 19 reactions were shown in the main text (*i.e.*, the 19 having at least one $\varphi_i > 2/L$), and those are enclosed in boxes within the figure below. A subset of these was discriminatory for metabosystems (see Text S3 for details), and they are enclosed by bold boxes in Figure 1 below. The full composition ribbon has 3 rows, one for each metabosystem specified in the model. Each row has 100 elements, representing 100 mixing probabilities that sum to 1.0. To facilitate its presentation, the full composition ribbon was divided into 5 subsets, which are stacked vertically within Figure 1 below.

Subsystems are labeled S1 to S100. Therefore, the first vertical set of three elements (having the same red color) represent the mixing probabilities of subnetwork S1 in each of the three different metabosystems. Taken in the vertical direction, the three values for S1 do not sum to 1.0. However, they do portray relative contributions. A long bar indicates a large contribution of a given subsystem to a metabosystem, and taken vertically the larger the difference in the length of each bar the larger the difference between metabosystems for that subnetworks. The ribbon diagram is intended only to provide a quick means of visualizing relative contributions of subsystems to metabosystesm; the actual mixing probabilities are provided in Data File S1.

BiomeNet revealed that the subsystems in Metabosystem 1 had a high contribution to carnivore samples and a low contribution to the herbivore samples (Figure 3 in main text). From the ribbon diagram in Figure 1 above, subnetwork S49 is highly discriminatory for Metabosystem 1. The EC number, substrates, products and mixing

probabilities for the principal reactions in mammalian subnetwork S49 are provided in Table S1.

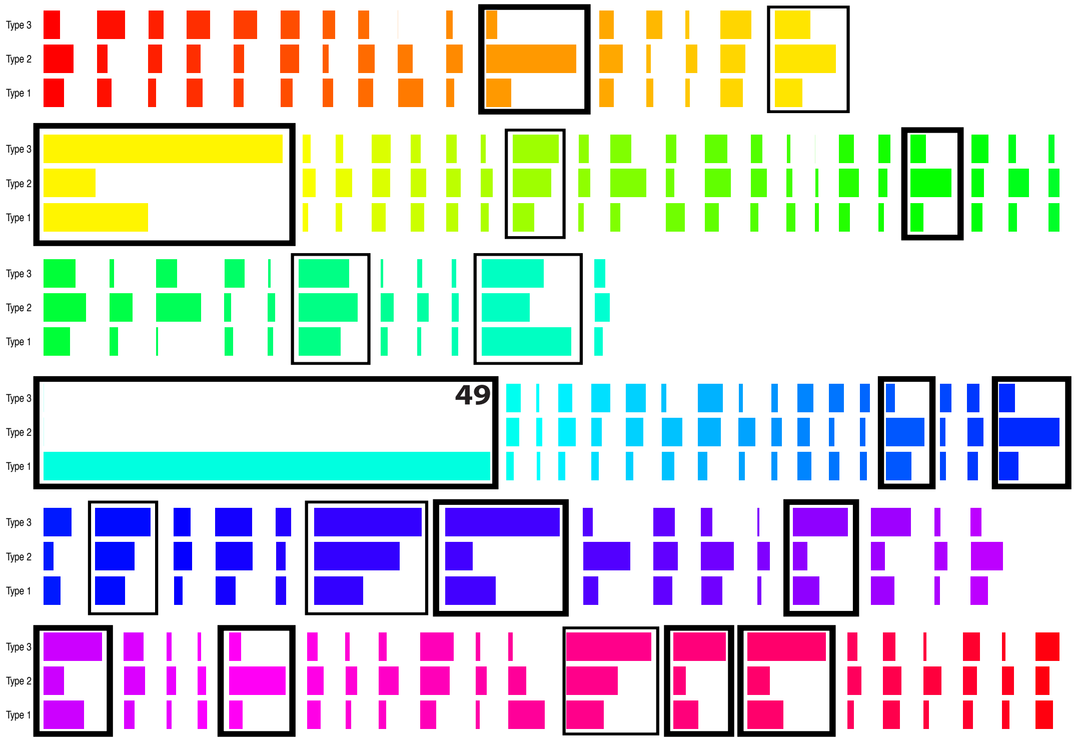

S7: Figure 1 Subnetwork composition ribbon plot for all 100 subnetworks in the mammal dataset. Subnetworks are numbered from 1 to 100 starting from the top row. Row one contains subnetworks S1-S16, row two contains S17-S37, row three contains subnetworks S38-S48, row four contains subnetworks S49-S65, row five S66-S80, row six S81-S100. The principal subnetworks are enlcosed in boxes, and the discriminatory subnetworks are enclosed in bold boxes. One subnetwork, S49, was highly discriminatory for metabosystem 1.
