# Supplementary material for: BiomeNet: A Bayesian Model for Inference of Metabolic Divergence among Microbial Communities
Source: PLoS Comput Biol. 2014 Nov 20;10(11):e1003918. doi: 10.1371/journal.pcbi.1003918 (PMC4238953; doi:10.1371/journal.pcbi.1003918)
Supplement: Text S8 — Subnetwork composition of the human IBD/healthy metabosystems. A presentation of the inferred contribution of 100 subnetworks to three metabosystems in the model, and the identification of the most discriminatory subnetworks for the metabosystem that was most associated with IBD (metabosystem 2). (PDF) [file pcbi.1003918.s008.pdf]

Text S8: Subnetwork composition of the human IBD/healthy metabosystems.

Figure 1 below shows the composition ribbon for all 100 subnetworks for the IBD/healthy individuals. Only 22 reactions were shown in the main text (*i.e.*, the 22 having at least one $\varphi_i > 2/L$), and those are enclosed in boxes within the figure below. A subset of these was discriminatory for metabosystems (see Text S3 for details), and they are enclosed by bold boxes in Figure 1 below. The full composition ribbon has 3 rows, one for each metabosystem specified in the model. Each row has 100 elements, representing 100 mixing probabilities that sum to 1.0. To facilitate its presentation, the full composition ribbon was divided into 6 subsets, which are stacked vertically within Figure 1 below.

Subsystems are labeled S1 to S100. Therefore, the first vertical set of three elements (having the same red color) represent the mixing probabilities of subnetwork S1 in each of the three different metabosystems. Taken in the vertical direction, the three values do not sum to 1.0. However, they do portray relative contributions. A long bar indicates a large contribution of a given subsystem to a metabosystem, and taken vertically the larger the difference in the length of each bar the bigger the difference between metabosystems for that subnetworks. The ribbon diagram is intended only to provide a quick means of visualizing relative contributions of subsystems to metabosystesm; the actual mixing probabilities are provided in Data File S1.

BiomeNet revealed an association between the subnetworks in Metabosystem 2 and the human IBD samples. From the ribbon diagram in 1 above, subnetworks S38, S64 and S73 can be seen to have a much higher contribution to Metabosystem 2 compared to the other metabosystems. The EC number, substrates, products and mixing

probabilities for the principal reactions subnetwork S38, S64 and S73 are provided in Tables S2, S3 and S4 respectively.

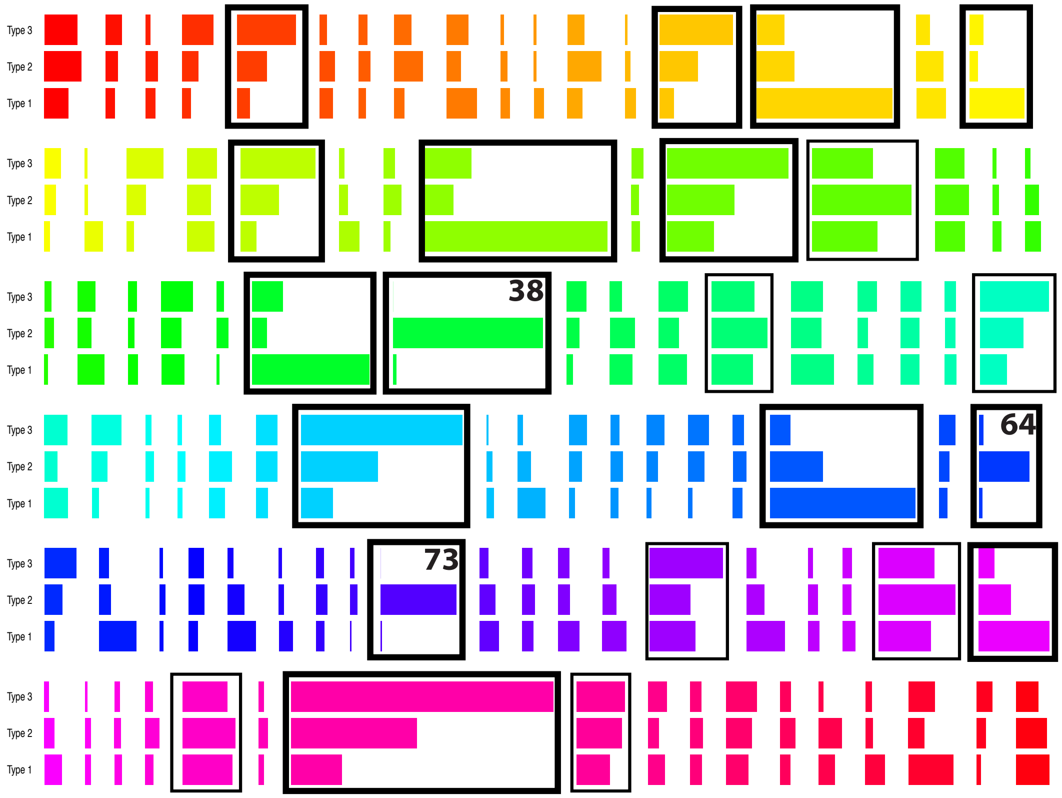

S8: Figure 1 Composition ribbon for all 100 subnetworks for the IBD/healthy data. Subnetworks are numbered from 1 to 100 starting from the top row. Row one contains subnetworks S1-S17, row two contains S18-S31, row three contains subnetworks S32-S47, row four contains subnetworks S48-S64, row five S65-S83, row six S84-S100.The predominat subnetworks are enlcosed in boxes, and the discriminatory subnetworks are enclosed in bold boxes. Three subnetworks (S38, S64 & S73) were highly discriminatory for metabosystem 2, which tends to have higher prevalance in IBD patients.
