# Supplementary material for: BiomeNet: A Bayesian Model for Inference of Metabolic Divergence among Microbial Communities
Source: PLoS Comput Biol. 2014 Nov 20;10(11):e1003918. doi: 10.1371/journal.pcbi.1003918 (PMC4238953; doi:10.1371/journal.pcbi.1003918)
Supplement: Text S9 — Core subnetworks. Definition and identification of core subnetworks under the BiomeNet modeling framework. (PDF) [file pcbi.1003918.s009.pdf]

Text S9: Core subnetworks.

We used BiomeNet to identify core subnetworks. Here, we consider subnetworks to be a part of the core set if they make a considerable contribution to the metabolic network of all samples. Moreover, we consider core subnetworks to be non-differentiating. Therefore, a subnetwork that contributes to all samples but with highly variable amounts in different individuals is not considered a core subnetwork.

One can argue that using a simple, but *ad hoc*, approach is sufficient to identify a core set of reactions. For instance, a relative count of reactions in each sample can be used to extract a list of reactions that are abundant across different samples. Our model however can be used to group reactions into subnetworks that are similarly prevalent in all samples. We choose to employ this model-derived definition of core subnetworks. This will have substantial benefits over simply having a list of core reactions. Such core subnetworks, resolved by grouping a much smaller number of reactions, can be interpreted and explained more easily. Connections between these core subnetworks can also be investigated, as these subnetworks can have overlapping components under BiomeNet. These subnetworks will lend themselves to better visualizations due to their high connectivity and smaller number of reactions.

Model-based definition of core subnetworks

BiomeNet estimates the metabosystem composition of each sample and the subnetwork composition of each metabosystem. These estimates can be used for determining the subnetwork composition of the metabolic network associated with each sample. If the metabosystem composition of sample i is given by θ_i and the subnetwork

composition of different metabosystems are given in matrix φ, then the subnetwork composition of sample i can be estimated by $\theta_i \times \varphi$. This will result in a vector of probability values with length L (number of subnetworks) that sum to one. Note that these memberships are with respect to samples as opposed to metabosystems.

To determine the core subnetworks we examine the membership of each subnetwork to all samples. If there were, say, 100 subnetworks and all those subnetworks were considered equally likely within a metabolic network, and then each subnetwork would have a 1% contribution to that network. However, under BiomeNet a metabosystem is typically characterized by a small subset of subnetworks with high contribution, and the remaining subnetworks having low contribution values (*i.e.*, <1% in this case). Thus, for L =100, we consider any subnetwork with a membership probability of greater than 0.01 to be a candidate core subnetwork. Among those subnetworks that have greater than 1% contribution in all samples, we examine the variance in the contributions across the samples and only consider those subnetworks with low contribution variance (relatively similar contribution across all samples) as contributing to the core.

Core subnetworks in the mammalian and human datasets.

For the mammal dataset, we fitted our model for three metabosystems and 100 subnetworks. Using the criterion above, we identified 9 subnetworks (16, 24, 43, 47,62, 67, 81, 92, and 94) as contributing to the core. Mapping the reactions found in these subnetworks shows connection between multiple KEGG pathways (Figure 1 below).

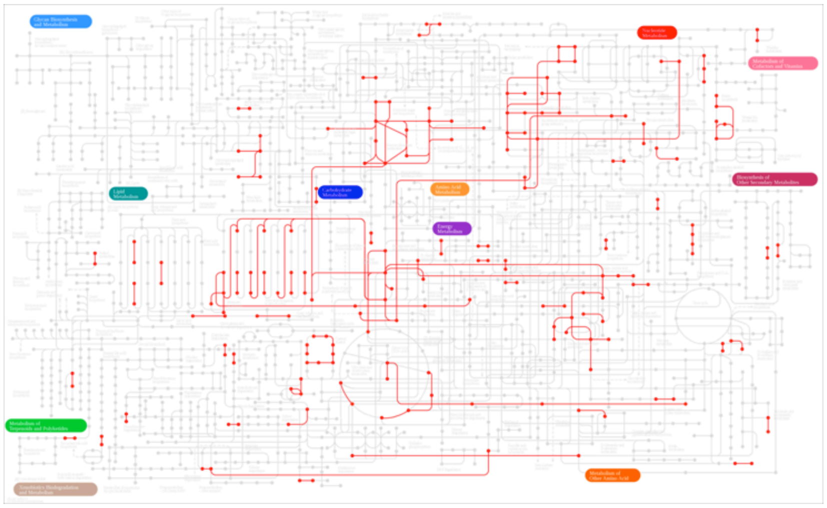

S8: Figure 1 Mapping of reactions in 9 core subnetworks of mammals to a KEGG metabolic cartography. Red lines indicate the reactions

For the IBD/healthy dataset, we trained our model for three metabosystems and 100 subnetworks. Using the same criteria, we identified 5 subnetworks (42, 43, 82, 88, and 91) as contributing the core. Mapping the reactions found in these subnetworks shows connection between multiple KEGG pathways (Figure 2 below).

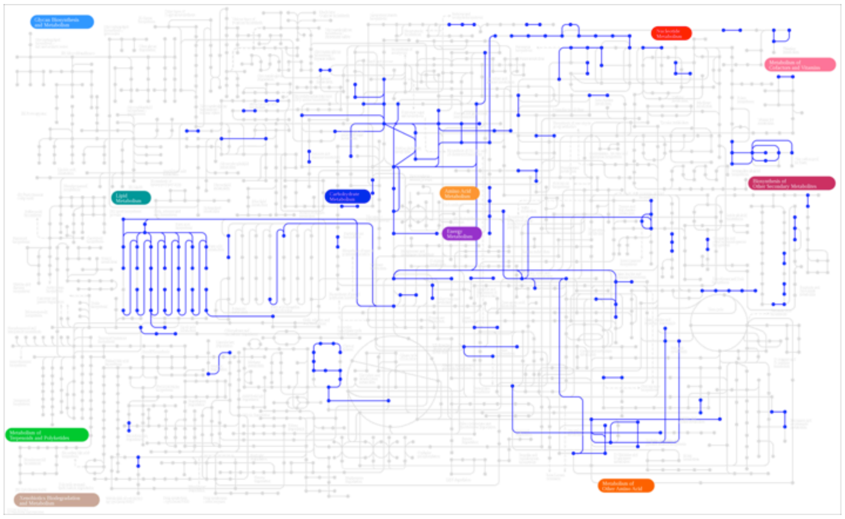

S8: Figure 2 Mapping of reactions in the 5 core subnetworks in humans to a KEGG metabolic cartography. Blue lines show the reactions found to be part of the core.
