# Supplementary material for: BiomeNet: A Bayesian Model for Inference of Metabolic Divergence among Microbial Communities
Source: PLoS Comput Biol. 2014 Nov 20;10(11):e1003918. doi: 10.1371/journal.pcbi.1003918 (PMC4238953; doi:10.1371/journal.pcbi.1003918)
Supplement: Table S1 — Composition of subnetwork 49 inferred from the mammalian dataset. The table gives the KEGG reaction numbers, substrates and products for the principal reactions in mammalian subnetwork 49. Because the model does not rigidly define subnetworks, each reaction in the dataset will have an estimated mixing probability. As the majority of reactions make only a trivial contribution to this subnetwork (nearly zero), we filtered out any reaction with a contribution less than 2/R, where R is the count of unique reactions summed over all the samples in a dataset. This resulted in a subset of 25 reactions having a posterior density>0.99. (PDF) [file pcbi.1003918.s010.pdf]

Table S1. Principal reactions of subnetwork 49 inferred from the mammalian dataset.

Reaction No.	Reactant Numbers	Pathway
**R00811**	C00089 + C04261 <=> C16688 + C00615	Starch and sucrose metabolism
**R02630**	C04261 + C00159 <=> C00615 + C00275	Fructose and mannose metabolism<br>Amino sugar and nucleotide sugar metabolism
**R02631**	C04261 + C00329 <=> C00615 + C00352	Amino sugar and nucleotide sugar metabolism
**R02704**	C04261 + C00392 <=> C00615 + C00644	Fructose and mannose metabolism
**R02738**	C04261 + C00031 <=> C00615 + C00668	Glycolysis / Gluconeogenesis<br>Amino sugar and nucleotide sugar metabolism
**R02780**	C01083 + C04261 <=> C00689 + C00615	Starch and sucrose metabolism
**R03076**	C04261 + C11477 <=> C00615 + C00934	general reaction
**R03232**	C04261 + C00095 <=> C00615 + C01094	Fructose and mannose metabolism
**R04076**	C04261 + C00247 <=> C00615 + C02888	Fructose and mannose metabolism
**R04111**	C04261 + C00208 <=> C00615 + C02995	Starch and sucrose metabolism
**R04393**	C04261 + C00243 <=> C00615 + C05396	Galactose metabolism
**R04394**	C04261 + C01451 <=> C00615 + C06188	Glycolysis / Gluconeogenesis
**R05132**	C04261 + C06186 <=> C00615 + C06187	Glycolysis / Gluconeogenesis
**R05199**	C04261 + C00140 <=> C00615 + C00357	Amino sugar and nucleotide sugar metabolism
**R05570**	C01697 + C04261 <=> C06311 + C00615	Galactose metabolism
**R05820**	C04261 + C00794 <=> C00615 + C01096	Fructose and mannose metabolism
**R07671**	C04261 + C00072 <=> C00615 + C16186	Ascorbate and aldarate metabolism
**R08367**	C02262 + C04261 <=> C06377 + C00615	Galactose metabolism
**R08559**	C02713 + C04261 <=> C16698 + C00615	Amino sugar and nucleotide sugar metabolism
**R08860**	C04261 + C01019 <=> C00615 + C02985	Amino sugar and nucleotide sugar metabolism
**R02628**	C00074 + C00615 <=> C00022 + C04261	enzyme I of the phosphotransferase system
**R03921**	C16688 + C00001 <=> C02336 + C00668	Starch and sucrose metabolism
**R08555**	C00256 + C00357 <=> C16698 + C00001	Amino sugar and nucleotide sugar metabolism
**R07677**	C16186 + C00001 <=> C14899	Ascorbate and aldarate metabolism
**R02087**	C00357 <=> C04257	Amino sugar and nucleotide sugar metabolism
