# Supplementary material for: BiomeNet: A Bayesian Model for Inference of Metabolic Divergence among Microbial Communities
Source: PLoS Comput Biol. 2014 Nov 20;10(11):e1003918. doi: 10.1371/journal.pcbi.1003918 (PMC4238953; doi:10.1371/journal.pcbi.1003918)
Supplement: Table S2 — Composition of subnetwork 38 inferred from the human dataset. The table gives the KEGG reaction numbers, substrates and products for the principal reactions in human subnetwork 38. Because the model does not rigidly define subnetworks, each reaction in the dataset will have an estimated mixing probability. As the majority of reactions make only a trivial contribution to this subnetwork (nearly zero), we filtered out any reaction with a contribution less than 2/R, where R is the count of unique reactions summed over all the samples in a dataset. This resulted in a subset of 29 reactions having a posterior density>0.99. (PDF) [file pcbi.1003918.s011.pdf]

Table S2 Principal reactions of subnetwork 38 inferred from the human dataset.

Reaction Number	Reactant Numbers	Pathway
**R00022**	C01674 + C00001 <=> 2 C00140	Amino sugar and nucleotide sugar metabolism
**R02630**	C04261 + C00159 <=> C00615 + C00275	Amino sugar and nucleotide sugar metabolism
**R02631**	C04261 + C00329 <=> C00615 + C00352	Amino sugar and nucleotide sugar metabolism
**R02704**	C04261 + C00392 <=> C00615 + C00644	Fructose and mannose metabolism
**R02738**	C04261 + C00031 <=> C00615 + C00668	Glycolysis / Gluconeogenesis<br>Amino sugar and nucleotide sugar metabolism
**R02780**	C01083 + C04261 <=> C00689 + C00615	Starch and sucrose metabolism
**R03232**	C04261 + C00095 <=> C00615 + C01094	Fructose and mannose metabolism
**R04076**	C04261 + C00247 <=> C00615 + C02888	Fructose and mannose metabolism
**R05199**	C04261 + C00140 <=> C00615 + C00357	Amino sugar and nucleotide sugar metabolism
**R05820**	C04261 + C00794 <=> C00615 + C01096	Fructose and mannose metabolism
**R08860**	C04261 + C01019 <=> C00615 + C02985	Amino sugar and nucleotide sugar metabolism
**R03076**	C04261 + C11477 <=> C00615 + C00934	General reaction
**R02568**	C01094 <=> C00111 + C00577	Fructose and mannose metabolism
**R02628**	C00074 + C00615 <=> C00022 + C04261	enzyme I of the phosphotransferase system
**R02623**	C00019 + C00614 <=> C00021 + C04142	(not KEGG pathway assignment)
**R03538**	C02355 + C00001 <=> C01368	Pyrimidine metabolism
**R07411**	C00032 + C00001 + C00448 <=> C15672 + C00013	Porphyrin and chlorophyll metabolism
**R01206**	C00461 + C00001 <=> C00140 + C00461	Amino sugar and nucleotide sugar metabolism
**R05207**	C05952 + C00025 <=> C06462 + C00001	(not KEGG pathway assignment)
**R06942**	C14144 + C00001 <=> C14145	Phenylalanine metabolism<br>Caprolactam degradation
**R04373**	C04755 + C00001 <=> C00140 + C04010	(not KEGG pathway assignment)
**R02624**	C04142 + C00001 <=> C00614 + C00132	(not KEGG pathway assignment)
**R04225**	C00671 + C15972 <=> C15979 + C00011	(not KEGG pathway assignment)
**R07603**	C00671 + C00068 <=> C15978 + C00011	Valine, leucine and isoleucine degradation
**R07604**	C15978 + C15972 <=> C15979 + C00068	Valine, leucine and isoleucine degradation

**R00310**	C02191 + C14818 <=> C00032 + 2 C00080	Porphyrin and chlorophyll metabolism
**R01330**	C00131 + C00159 <=> C00206 + C00275	(not KEGG pathway assignment)
**R01965**	C00131 + C00329 <=> C00206 + C00352	(not KEGG pathway assignment)
**R02868**	C00131 + C00794 <=> C00206 + C01096	(not KEGG pathway assignment)
