# Supplementary material for: BiomeNet: A Bayesian Model for Inference of Metabolic Divergence among Microbial Communities
Source: PLoS Comput Biol. 2014 Nov 20;10(11):e1003918. doi: 10.1371/journal.pcbi.1003918 (PMC4238953; doi:10.1371/journal.pcbi.1003918)
Supplement: Table S3 — Composition of subnetwork 64 inferred from the human dataset. The table gives the KEGG reaction numbers, substrates and products for the principal reactions in human subnetwork 64. Because the model does not rigidly define subnetworks, each reaction in the dataset will have an estimated mixing probability. As the majority of reactions make only a trivial contribution to this subnetwork (nearly zero), we filtered out any reaction with a contribution less than 2/R, where R is the count of unique reactions summed over all the samples in a dataset. This resulted in a subset of 19 reactions having a posterior density>0.99. (PDF) [file pcbi.1003918.s012.pdf]

Table S3 Principal reactions of subnetwork 64 inferred from the human dataset.

Reaction Number	Reactant Numbers	Pathway
**R04394**	C04261 + C01451 <=> C00615 + C06188	Glycolysis / Gluconeogenesis
**R05570**	C01697 + C04261 <=> C06311 + C00615	Galactose metabolism
**R07671**	C04261 + C00072 <=> C00615 + C16186	Ascorbate and aldarate metabolism
**R05636**	C00022 + C00118 <=> C11437 + C00011	Thiamine metabolism<br>Terpenoid backbone biosynthesis
**R07677**	C16186 + C00001 <=> C14899	Ascorbate and aldarate metabolism
**R05688**	C11434 + C00006 <=> C11437 + C00005 + C00080	Terpenoid backbone biosynthesis
**R07662**	C16156 + C00001 <=> C16157 + C00058	
**R08192**	C16179 + C00001 <=> C00010 + C16522	Biosynthesis of unsaturated fatty acids
**R09185**	C18272 <=> C18281 + C00058	Polycyclic aromatic hydrocarbon degradation
**R05134**	C06188 + C00001 <=> C02323 + C01172	Glycolysis / Gluconeogenesis
**R05046**	C05922 + C00001 <=> C05923 + C00058	Folate biosynthesis
**R02940**	C05985 + C00003 + C00001 <=> C00804 + C00004 + C00080	Propanoate metabolism
**R05838**	C11638 + C11437 <=> C00627 + C00009 + 2 C00001	Vitamin B6 metabolism
**R03076**	C04261 + C11477 <=> C00615 + C00934	general reaction
**R04488**	C04484 + C00001 <=> C06035 + C00058	Benzoate degradation
**R05608**	C00879 <=> C00679 + C00001	Ascorbate and aldarate metabolism
**R02754**	C00679 <=> C00022 + C01146	Ascorbate and aldarate metabolism
**R04489**	C05375 <=> C04484	
**R00653**	C03145 + C00001 <=> C00058 + C00073	Cysteine and methionine metabolism<br>Glyoxylate and dicarboxylate metabolism
