# Supplementary material for: BiomeNet: A Bayesian Model for Inference of Metabolic Divergence among Microbial Communities
Source: PLoS Comput Biol. 2014 Nov 20;10(11):e1003918. doi: 10.1371/journal.pcbi.1003918 (PMC4238953; doi:10.1371/journal.pcbi.1003918)
Supplement: Table S4 — Composition of subnetwork 73 inferred from the human dataset. The table gives the KEGG reaction numbers, substrates and products for the principal reactions in human subnetwork 73. Because the model does not rigidly define subnetworks, each reaction in the dataset will have an estimated mixing probability. As the majority of reactions make only a trivial contribution to this subnetwork (nearly zero), we filtered out any reaction with a contribution less than 2/R, where R is the count of unique reactions summed over all the samples in a dataset. This resulted in a subset of 18 reactions having a posterior density>0.99. (PDF) [file pcbi.1003918.s013.pdf]

Table S4. Principal reactions of subnetwork 73 inferred from the human dataset.

Reaction Number	Reactant Numbers	Pathway
**R00811**	C00089 + C04261 <=> C16688 + C00615	Starch and sucrose metabolism
**R04111**	C04261 + C00208 <=> C00615 + C02995	Starch and sucrose metabolism
**R04393**	C04261 + C00243 <=> C00615 + C05396	Galactose metabolism
**R05132**	C04261 + C06186 <=> C00615 + C06187	Glycolysis / Gluconeogenesis
**R08367**	C02262 + C04261 <=> C06377 + C00615	Galactose metabolism
**R06178**	C05893 + C11826 <=> C04574 + C11826	
**R06729**	C12448 + C00001 <=> C10858 + C00132	Tropane, piperidine and pyridine alkaloid biosynthesis
**R06728**	C01416 + C00001 <=> C12448 + C00180	Tropane, piperidine and pyridine alkaloid biosynthesis
**R03921**	C16688 + C00001 <=> C02336 + C00668	Starch and sucrose metabolism
**R05133**	C06187 + C00001 <=> C00530 + C01172	Glycolysis / Gluconeogenesis
**R03076**	C04261 + C11477 <=> C00615 + C00934	general reaction
**R01424**	C01586 + C00001 <=> C00180 + C00037	Phenylalanine metabolism
**R01421**	C06206 + C00001 <=> C00180 + C00009	Aminobenzoate degradation
**R05055**	C05951 + C00001 <=> C05952 + C00037	Arachidonic acid metabolism
**R05591**	C09814 + 2 C00001 <=> C00180 + C00014	Aminobenzoate degradation
**R02933**	C01040 + C00001 <=> C00800	Ascorbate and aldarate metabolism
**R05590**	C09815 + C00001 <=> C00180 + C00014	Aminobenzoate degradation
**R02739**	C00668 <=> C01172	Glycolysis / Gluconeogenesis<br>Pentose phosphate pathway
